# Chemically Anchored PbS‐2PACz CQDs Inks for Scalable HTL in Narrow‐Bandgap and All‐Perovskite Tandem Solar Cells

**DOI:** 10.1002/smll.202505059

**Published:** 2025-09-27

**Authors:** Seung Hwa Hong, Sangheon Lee, Sunwoo Kim, Jaegwan Jung, Doeun Shim, Eunhye Cho, Minwoo Jeong, Mahnmin Choi, Taewon Goo, Jugyoung Kim, Sang Jun Park, Meeree Kim, Jae Ryoung Lee, Gabeen Cho, Sangwook Lee, Yong‐Hyun Kim, Dong Hoe Kim, Sohee Jeong

**Affiliations:** ^1^ Department of Energy Science (DOES) Sungkyunkwan University (SKKU) Suwon 16419 Republic of Korea; ^2^ Department of Materials Science and Engineering Korea University Seoul 02841 Republic of Korea; ^3^ School of Materials Science and Engineering Kyungpook National University Daegu 41566 Republic of Korea; ^4^ Department of Physics Korea Advanced Institute of Science and Technology (KAIST) Daejeon 34141 Republic of Korea; ^5^ Graduate School of Semiconductor Technology Korea Advanced Institute of Science and Technology (KAIST) Daejeon 34141 Republic of Korea; ^6^ School of Physics Institute of Science Suranaree University of Technology Nakhon Ratchasima 30000 Thailand; ^7^ Department of Future Energy Engineering (DFEE) Sungkyunkwan University (SKKU) Suwon 16419 Republic of Korea; ^8^ Sungkyunkwan Institute of Energy Science and Technology (SIEST) Suwon 16419 Republic of Korea

**Keywords:** all‐perovskite tandem solar cells, colloidal quantum dots, hole transport layer, interface engineering, narrow‐bandgap perovskite solar cells

## Abstract

Mixed‐metal narrow‐bandgap (NBG) Sn‐Pb perovskites are essential for achieving high‐efficiency all‐perovskite tandem solar cells **(APTSCs)**. However, their single‐junction performance is limited by interfacial recombination and inefficient charge extraction, due to non‐uniform hole transport layers (HTLs). Here, PbS‐2PACz colloidal quantum dots (CQDs) are synthesized, chemically anchoring [2‐(9H‐carbazol‐9‐yl)ethyl]phosphonic acid (2PACz) onto PbS CQDs via solution‐phase ligand exchange. The resulting PbS‐2PACz CQD ink demonstrates excellent colloidal stability in weakly polar solvents and yields uniform, defect‐suppressing films on perovskites. To further enhance the perovskite/HTL interface, an additional 2PACz treatment deepens the valence band (−5.60 eV), reduces trap density, and optimizes energy‐level alignment. Consequently, NBG perovskite solar cells utilizing PbS‐2PACz with additional 2PACz achieve power conversion efficiencies (PCEs) of 22.84% ± 0.55, exceeding devices using poly(3,4‐ethylenedioxythiophene) polystyrene sulfonate (PEDOT:PSS) and 2PACz self‐assembled monolayers (SAMs). Integrated into APTSCs with a 1.77 eV wide‐bandgap top cell, PbS‐2PACz achieves a PCE of 25.05% ± 0.41, significantly outperforming PEDOT:PSS‐based tandems (21.60% ± 0.91). This work highlights PbS‐2PACz as an effective HTL material that enhances hole extraction, reproducibility, and scalability for high‐performance perovskite solar cells.

## Graphical abstract

PbS‐2PACz colloidal‐quantum‐dot ink, prepared via solution‐phase ligand exchange, forms conformal and defect‐suppressing interlayers. Subsequent treatment with a [2‐(9H‐carbazol‐9‐yl)ethyl]phosphonic acid (2PACz) solution yields a saturated PbS‐2PACz film with enhanced 2PACz coverage on the PbS surface. These HTLs enable favorable energy‐level alignment, reduced interfacial trap density, and efficient hole extraction, leading to high efficiency and reproducibility in narrow‐bandgap and all‐perovskite tandem solar cells.

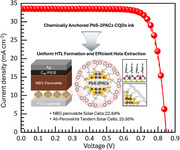

## 1 Introduction

Perovskite solar cells (PSCs) have emerged as a transformative photovoltaic system because of their exceptional optical absorption, tunable bandgaps, and high tolerance to defects.^[^ ^1^, ^2^, ^3^ ^]^ In particular, Sn–Pb mixed perovskites are regarded as promising narrow‐bandgap (NBG) absorbers for both single‐junction and all‐perovskite tandem solar cells (APTSCs), due to their tunable bandgaps (1.2–1.4 eV) and extended absorption in the near‐infrared region.^[^ ^4^, ^5^, ^6^, ^9^ ^]^ However, despite rapid progress yielding power conversion efficiencies (PCEs) above 23% in single‐junction devices, Sn–Pb perovskites still underperform compared to their Pb‐only counterparts (27%), mainly due to interfacial recombination losses.^[^ ^7^ ^,^ ^8^ ^]^

Specifically, energy‐level misalignment and poor contact at the perovskite/hole transport layer (HTL), especially in Sn‐rich compositions, where the shallow valence band leads recombination pathways.^[^ ^10^, ^11^ ^]^ These challenges impose the need for advanced HTLs that provide conformal surface coverage, strong electronic coupling, and energetically favorable alignment with the Sn–Pb PSCs.^[^ ^12^, ^13^ ^]^ Conventional HTLs such as spin‐coated poly(3,4‐ethylenedioxythiophene) polystyrene sulfonate (PEDOT:PSS) or self‐assembled monolayers (SAMs) like [2‐(9H‐carbazol‐9‐yl)ethyl]phosphonic acid (2PACz), have been widely applied in p‐i‐n NBG PSCs.^[^ ^14^, ^15^, ^16^, ^17^ ^]^ Still, PEDOT:PSS is highly acidic and hygroscopic thus leads device degradation.^[^ ^18^, ^19^, ^20^ ^]^ In the case of 2PACz SAM layer, non‐uniform coverage with holes on bottom layer often cause interfacial recombination thus reducing the collection efficiency.^[^ ^21^, ^22^, ^23^ ^]^ Such non‐uniformity is primarily attributed to micelle aggregation induced by the amphiphilic character of 2PACz, which hinders the formation of a uniform and densely packed monolayer.^[^ ^24^, ^25^ ^]^ The HTL having chemical robustness and processing compatibility to form conformal and defect‐tolerant interfaces is in need for realizing the high‐performance perovskite photovoltaics.

To address the limitations of previously explored HTLs in NBG or tandem PSCs, we present a multiscale assembly strategy based on the PbS–2PACz system. PbS colloidal quantum dots (CQDs) not only serve as excellent nanoscale scaffolds, but also function as p‐type semiconductors with tunable energy levels and outstanding solution processability.^[^ ^26^, ^27^, ^28^, ^29^, ^30^ ^]^ While PbS CQDs functionalized with various ligands have been previously investigated as HTLs in solar cells, these films are typically require polar solvents during the processing, posing significant risks of damaging underlying perovskite layers, especially in tandem architectures with solvent‐sensitive subcells.^[^ ^31^ ^]^ In this study, we synthesized 2PACz decorated PbS CQDs having excellent dispersibility in weakly polar solvents, ensuring solvent orthogonality essential for tandem fabrication involving polar‐solvent‐sensitive perovskites. Prepared PbS‐2PACz CQD inks are introduced as novel HTL materials for high‐efficiency NBG PSCs. Through pre‐coordination of 2PACz phosphonic acid groups with surface Pb, PbS–2PACz enables uniform and stable anchoring prior to film deposition. These strong interfacial interactions further promote favorable *π*–*π* stacking between adjacent carbazole groups on the (111) facets of PbS CQDs, as supported by X‐ray photoelectron spectroscopy (XPS), photoluminescence (PL), and density functional theory (DFT) calculation analyses. A secondary 2PACz coating on PbS‐2PACz film further saturated the surface and improved the interfacial passivation. NBG PSCs employing saturated PbS‐2PACz achieve a PCE of 22.84%, with significant improvements in open‐circuit voltage (*V_oc_*) and fill factor (FF) over PEDOT:PSS (18.40%) and conventional 2PACz SAMs (21.32%). When integrated into APTSCs, saturated PbS‐2PACz enabled a PCE of 25.05%, markedly higher than the 21.60% obtained with PEDOT:PSS.

## 2 Results and Discussion

### 2.1 Tailored PbS‐2PACz Inks: From Ligand Exchange to Electronic and Charge Transport Properties

**Figure 1:**
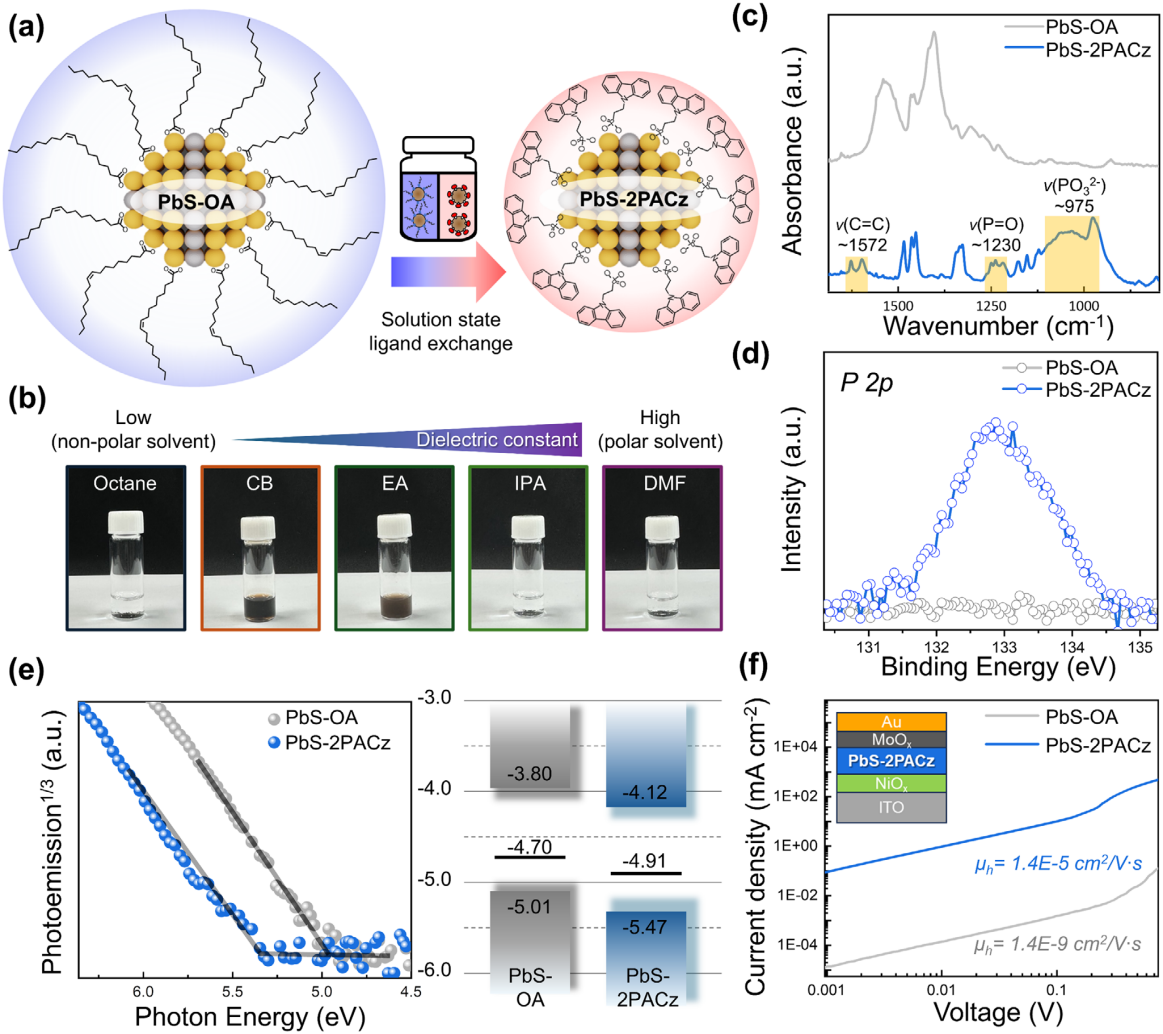
a) Schematic of PbS‐2PACz showing interaction between 2PACz ligands and PbS surface via solution‐phase ligand exchange. b) Photographs of PbS‐2PACz CQDs in various solvents, demonstrating excellent solubility in chlorobenzene. c) FT‐IR spectra before (gray) and after (blue) ligand exchange, confirming complete OA removal. d) XPS profiles (P 2p region) verifying incorporation of phosphonic acid ligand. e) APS profiles for determining VBM of PbS‐2PACz and PbS‐OA. (f) SCLC data for PbS‐2PACz, acquired using ITO/NiO_x_/PbS CQDs/MoO_x_/Au device configuration.

**Figure** **1**a provides a schematic of the 2PACz ligand assembly on the PbS (111) surface. As shown in Figure S1 (Supporting Information), the spectrum of the synthesized PbS CQDs exhibited an excitonic absorption peak at ≈850 nm (≈3 nm in diameter), indicating a predominantly (111)‐facet‐dominated truncated octahedral morphology.^[^ ^29^ ^]^ This structure provides a high‐energy, polar surface that not only facilitates strong 2PACz coordination but also enables effective passivation of the structure, unlike the relatively neutral (100) facets, which exhibit weaker binding tendencies. PbS‐2PACz was synthesized by replacing the long‐chain oleic acid (OA) ligands with 2PACz via solution‐state ligand exchange in a nitrogen‐filled glove box. This process minimizes oxidation and preserves the properties of the materials. After brief mixing and multiple washing steps, the purified PbS‐2PACz CQDs were obtained in powder form for integration into the device.

In addition to structural passivation, solvent compatibility is also crucial in the fabrication of perovskite‐based devices. Because perovskite layers are typically processed using highly polar solvents such as dimethylformamide (DMF) and dimethyl sulfoxide (DMSO), the underlying HTL must exhibit solvent orthogonality to avoid interfacial degradation during the sequential deposition steps. PbS‐2PACz satisfies this requirement as it is well dispersed in weakly polar solvents (e.g., chlorobenzene) while remaining insoluble in polar solvents (Figure 1b). This property enables damage‐free deposition of perovskite layers on the PbS‐2PACz film. Plausibly, the phosphonic acid‐anchoring groups in 2PACz form strong chemical bonds with the PbS surface, whereas the hydrophobic carbazole moieties minimize the interactions with polar solvents, thereby ensuring solvent orthogonality during solution processing. Additionally, the PbS–2PACz film exhibited a water contact angle of 72°, slightly lower than that of the PbS–OA film (89°), indicating the successful binding of hydrophilic phosphonic acid groups (Figure S2, Supporting Information).^[^ ^32^ ^]^ This enhanced solvent compatibility enabled sequential solution processing, preserving the integrity of the PbS interface while facilitating the formation of high‐quality perovskite layers.

To confirm the successful incorporation of 2PACz into the PbS CQDs, the samples were systematically analyzed using Fourier‐transform infrared (FT‐IR) spectroscopy, XPS, and ambient photoemission spectroscopy (APS). As shown in Figure 1c and S3 (Supporting Information), the FT‐IR spectrum of the PbS–2PACz film exhibits distinct P═O stretching (≈1230 cm^−1^) and PO_3_^2−^ vibrations (≈975 cm^−1^), along with the aromatic C═C stretching vibration of the carbazole moiety (≈1547 cm^−1^), clearly indicating the attachment of 2PACz and the removal of native oleic acid (OA) ligands. Notably, the P–O─H stretching modes at 945 and 1030 cm^−1^, readily observed in the spectrum of 2PACz, are absent in the PbS–2PACz film. This disappearance suggests that the phosphonic acid groups of 2PACz primarily coordinate with surface Pb^2^⁺ ions in a bi‐ or tridentate mode, rather than forming weak monodentate linkages.^[^ ^33^ ^]^ Monodentate coordination typically leaves a free P─OH group, which would give rise to detectable P─O─H stretching bands; the absence of these features strongly supports multidentate binding of the phosphonic groups to surface Pb.

Moreover, XPS analysis further supports this transformation, as the distinct P 2p peak observed in the spectrum of the PbS‐2PACz film (Figure 1d) was absent in that of the PbS‐OA film, confirming the successful incorporation of 2PACz. The APS data in Figure 1e illustrate the effect of 2PACz on the electronic‐band structure. The threshold energy was determined from the intersection of the linear extrapolation with the baseline.^[^ ^22^ ^]^ The valence band maximum (VBM) shifted from −5.01 eV (PbS‐OA) to −5.47 eV (PbS‐2PACz), attributed to the formation of an interfacial dipole induced by the phosphonic acid groups of 2PACz. Additionally, the higher electrostatic potential of 2PACz (2.00 Debye) compared to that of OA (1.18 Debye) contributes to this downward shift in the energy levels.^[^ ^16^, ^34^ ^]^ To further investigate the hole transport behavior, the hole mobility was evaluated through space‐charge‐limited current (SCLC) measurements using hole‐only devices (glass/indium tin oxide (ITO)/nickel oxide (NiO_x_)/PbS CQDs/molybdenum oxide (MoO_x_)/Au). The mobility was extracted based on the Mott‐Gurney law, as described by Equation S1 (Supporting Information). For the device incorporating the PbS‐2PACz film, the estimated film thickness was ≈30 nm, yielding a hole mobility of μ_*h*_ =  1.4 × 10^−5^ cm^2^ V^−1^s^−1^. In contrast, the PbS‐OA film afforded a significantly lower hole mobility of μ_*h*_ =  1.4 × 10^−9^ cm^2^ V^−1^s^−1^ (Figure 1f). This drastic difference is attributed to the long insulating alkyl chains in OA, which hinder electronic coupling and efficient charge transport.^[^ ^35^ ^]^

### 2.2 Hole Extraction and Uniform Film Formation with PbS‐2PACz in NBG PSCs

**Figure 2:**
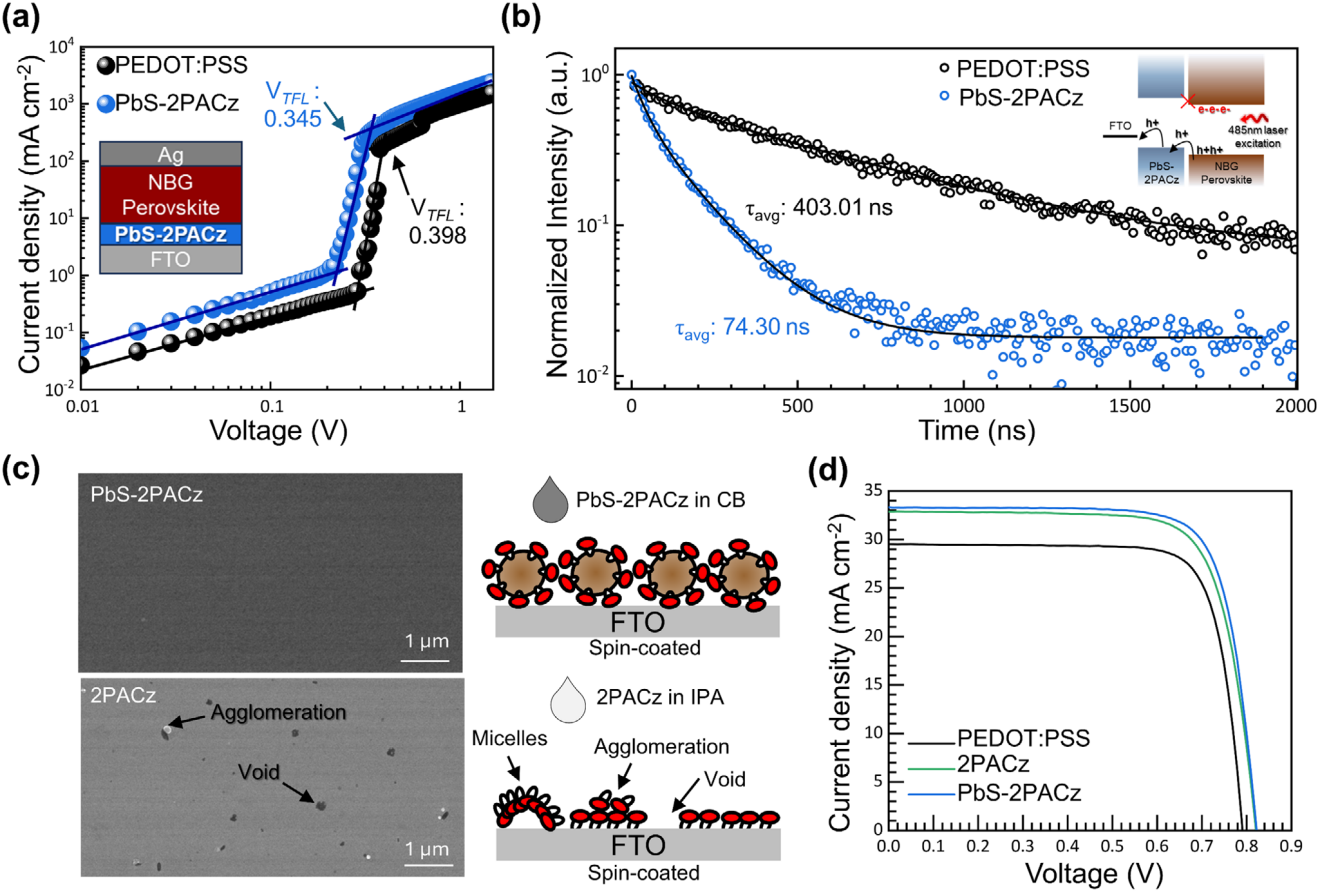
a) SCLC data for HTL devices (device configuration: ITO/HTL/NBG PSCs/Ag). PbS‐2PACz exhibits a lower *V*_TFL_, indicating reduced trap‐state density and enhanced charge transport. b) Normalized TR‐PL decay (fitted to bi‐exponential function) of NBG PSCs films on PEDOT:PSS and PbS‐2PACz, demonstrating enhanced hole extraction and improved carrier dynamics with PbS‐2PACz. c) SEM images and schematic of FTO/PbS‐2PACz and FTO/ 2PACz surfaces. PbS‐2PACz forms a uniform, pinhole‐free layer, whereas 2PACz film offers poor coverage with agglomeration and voids. d) *J–V* characteristics of NBG PSCs employing PEDOT:PSS, 2PACz, and PbS‐2PACz.

To investigate the hole extraction characteristics of the PbS‐2PACz film as an HTL under the perovskite layer, SCLC analysis was performed using hole‐only devices (glass/fluorine‐doped tin oxide (FTO)/HTL/NBG perovskite/Ag). For comparison, the PEDOT:PSS‐based reference devices were also analyzed. To ensure statistical relevance and reproducibility, three identically fabricated devices were measured for each configuration. The SCLC analysis revealed that the PbS‐2PACz‐based devices exhibited a lower trap‐filled limit voltage (*V*_TFL_) of 0.345 V, compared to 0.398 V for the PEDOT:PSS‐based devices (**Figure** **2**a; Figure S4, Supporting Information), indicating a significantly reduced density of charge‐trapping states. The trap density (*N_trap_*) was calculated using Equation S2 (Supporting Information), confirming that PbS‐2PACz exhibited a lower trap density (1.09 ± 0.02 × 10^15^ cm^−3^) compared to PEDOT:PSS (1.27 ± 0.01 × 10^15^ cm^−3^), further supporting the superiority of the former for defect passivation. Additionally, the dark current density‒voltage (*J–V)* measurements (Figure S5, Supporting Information) showed a higher offset voltage of 0.58 V for the PbS‐2PACz‐based devices, compared to 0.44 V for the PEDOT:PSS‐based devices, suggesting enhanced shunt resistance in the former. This improvement aligns with the lower trap density observed in the SCLC analysis, confirming the effective suppression of interfacial defects and enhanced charge‐transport properties of the PbS‐2PACz film.

Time‐resolved photoluminescence (TR‐PL) measurements were performed to further evaluate the hole‐extraction properties of the film (Figure 2b). The TR‐PL data were analyzed using a biexponential decay function, consisting of a fast decay lifetime (*τ*_1_), assigned to trap‐mediated non‐radiative recombination, and a slow decay lifetime (*τ*_2_), which correlated to radiative recombination of the free charge carriers before charge collection.^[^ ^36^ ^]^ The relevant parameters are listed in Table S1 (Supporting Information). The fast and slow decay lifetimes were both shorter in the case of PbS‐2PACz (*τ*_1_: 31.12 ns and *τ*_2_: 114.24 ns), where the fast decay process accounted for a larger fraction of the overall decay (A_1_: 0.48 and A_2_: 0.52). In contrast, for PEDOT:PSS, the fast and slow decay lifetimes were relatively longer (*τ*_1_: 135.48 ns and *τ*_2_: 560.54 ns), with the slow decay process accounting for a larger fraction of the overall decay, indicating that radiative recombination dominates over efficient charge transfer (A_1_: 0.37 and A_2_: 0.64). Thus, the average carrier lifetime (τ_avg_) declined steeply from 403.01 ns for PEDOT:PSS to 74.30 ns for PbS‐2PACz. These findings demonstrate that the device with PbS‐2PACz facilitates faster charge transfer across the perovskite/HTL interface while suppressing recombination pathways, thereby enhancing the overall charge extraction efficiency compared to that of PEDOT:PSS‐based devices.

To evaluate the film quality, the surface morphologies of PbS‐2PACz and pristine 2PACz were compared. Scanning electron microscopy (SEM) was used to directly observe the morphological distinctions, and a schematic of the respective configurations on FTO substrates is presented in Figure 2c. The surface of the PbS‐2PACz film was highly uniform, smooth, and pinhole‐free. This superior morphology is attributed to the strong interaction between 2PACz and the PbS CQDs, which ensures proper dispersion in the solvent, prevents micelle formation, and promotes the formation of a smooth, continuous film. This enhances the surface coverage, independent of the substrate roughness. In contrast, the spin‐coated 2PACz layers did not completely cover the surface, leading to voids and agglomeration.^[^ ^37^, ^38^ ^]^ This non‐uniform surface arises from the limited solubility of 2PACz, where the critical micelle concentration of 2PACz in 2‐propanol (IPA) induces aggregation during deposition.^[^ ^39^ ^]^ PbS‐2PACz provides ordering and thus facilitates the formation of a uniform morphology, regardless of the substrate type.

To validate the potential of the PbS‐2PACz film as an HTL for NBG PSCs, single‐junction p‐i‐n devices were fabricated; the *J–V* characteristics are graphically represented in Figure 2d. For comparison, devices were also prepared with PEDOT:PSS or spin‐coated 2PACz layers as the HTL, and the corresponding photovoltaic parameters are listed in Table S2 (Supporting Information). As expected, the PCE of the PSCs incorporating the PbS‐2PACz film 21.05% (*J_sc_* = 33.29 mA cm^−2^, *V_oc_* = 0.82 V, and FF = 76.85) was significantly enhanced compared to that of the PEDOT:PSS‐based PSCs (PCE of 18.40%, *J_sc_* = 29.52 mA cm^−2^, *V_oc_* = 0.79 V, and FF = 78.85). However, despite its superior morphology, the *V_oc_* and FF values remained similar to those of the spin‐coated 2PACz‐based PSCs (PCE of 21.32%, *J_sc_* = 33.07 mA cm^−2^, *V_oc_* = 0.84 V, and FF = 76.41), suggesting that the hole‐extraction capacity was quickly saturated with the existing 2PACz coverage.

### 2.3 Saturated 2PACz on PbS Surface for Trap Passivation, Interfacial Optimization, and Energy‐Level Alignment

**Figure 3:**
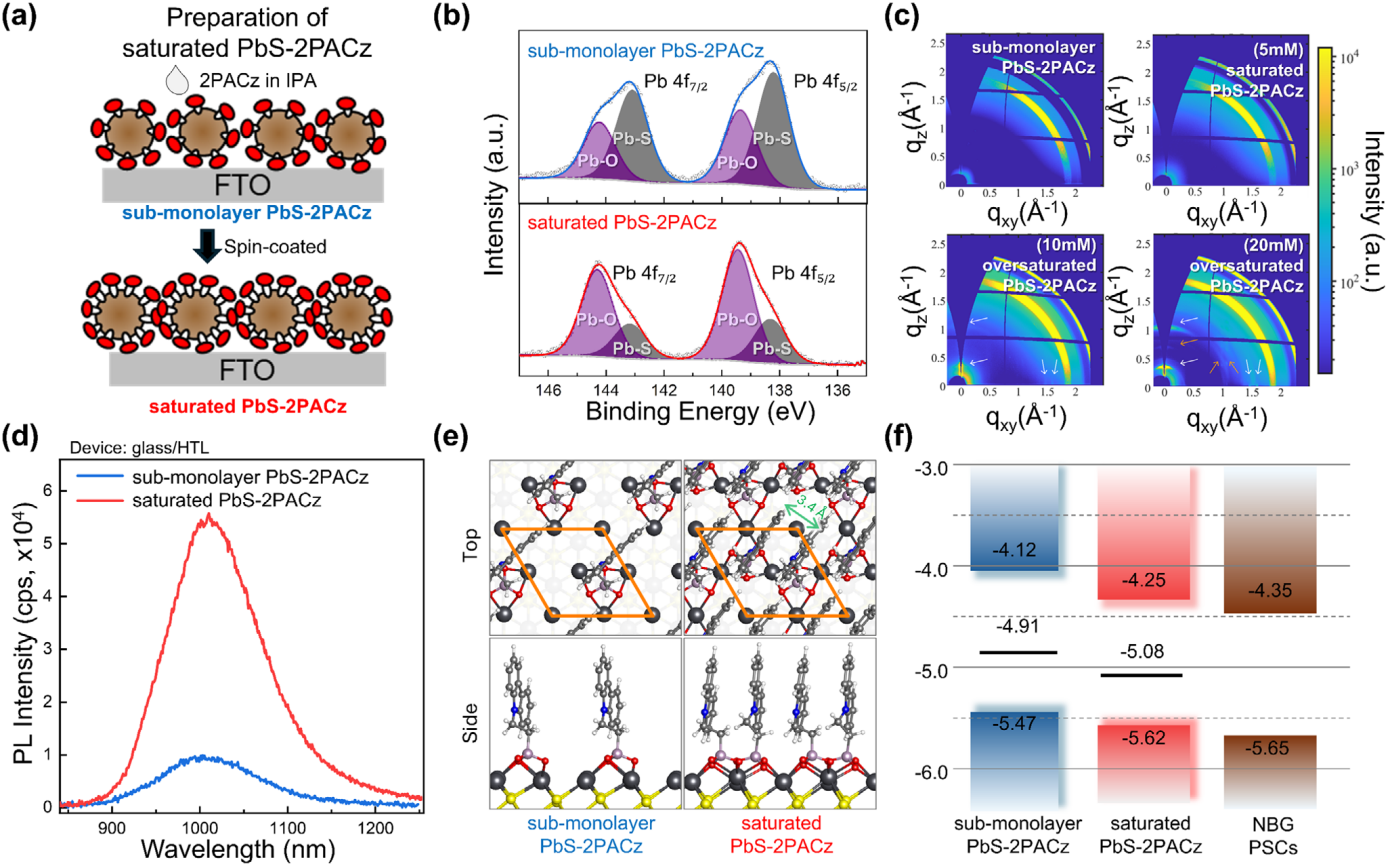
a) Schematic of additional 2PACz solution on sub‐monolayer PbS‐2PACz film for preparation of saturated PbS‐2PACz film. b) High‐resolution XP spectra (Pb 4f region) of PbS‐2PACz films before and after introducing additional 2PACz solution, showing shifts in Pb─S and Pb─O bonding peaks, indicating strengthened interfacial interactions. c) GIWAXS patterns of sub‐monolayer PbS‐2PACz with varying 2PACz concentrations (0 to 20 mm). d) ss‐PL data for sub‐monolayer PbS‐2PACz and saturated PbS‐2PACz films (device configuration: glass/HTL), confirming enhanced trap‐state passivation with increased 2PACz coverage. e) Top‐ and side‐view of atomic structure of PbS (111) surface passivated with a single 2PACz molecule (sub‐monolayer PbS‐2PACz) and with double 2PACz molecules (saturated PbS‐2PACz) in 2 × 2 unit cell indicated by orange solid line. In the double 2PACz configuration, the interplanar distance between the carbazole units is 3.4 Å, consistent with the well‐known π–π stacking distance, indicating a closely packed arrangement (Color legend: Pb (black), S (yellow), C (gray), H (white), N (blue), P (light purple), O (red)). f) Energy‐level diagram comparing sub‐monolayer PbS‐2PACz and saturated PbS‐2PACz with the perovskite layer, illustrating the impact of additional 2PACz on energy‐level alignment.

The above data, along with the TR‐PL results, indicate that the as‐prepared PbS‐2PACz film, hereafter referred to as sub‐monolayer PbS‐2PACz, provides only partial surface coverage by the 2PACz ligands. This limited coverage is likely insufficient for optimal passivation of the interface and energy‐level alignment, thereby constraining improvements in the *V_oc_* and FF. This limitation is attributed to the steric hindrance among the 2PACz molecules during the ligand‐exchange process, which prevented the formation of a fully packed PbS surface. The resulting undercoordinated Pb sites act as trap states, facilitating non‐radiative recombination.^[^ ^40^ ^]^ To overcome this limitation, the PbS‐2PACz films were systematically categorized as follows based on the surface coverage by 2PACz: sub‐monolayer PbS‐2PACz (initial ligand‐exchanged film), saturated PbS‐2PACz (spin‐coated with an optimized amount of 2PACz solution), and oversaturated PbS‐2PACz (with an excess amount of 2PACz solution). A schematic of the preparation strategy is shown in **Figure** **3**a.

This approach increased the surface density of the 2PACz molecules on the PbS surface, resulting in a more compact interfacial structure. To confirm the incorporation of additional 2PACz molecules, the surface of PbS was analyzed using XPS; the Pb 4f XPS profiles are presented in Figure 3b, and the corresponding binding energies (BEs) are listed in Table S3 (Supporting Information). The Pb 4f peaks associated with Pb–O coordination were observed at 144.3 eV (4f_5_/_2_) and 139.4 eV (4f_7_/_2_), whereas those corresponding to Pb–S coordination were located at 143.2 eV (4f_5_/_2_) and 138.4 eV (4f_7_/_2_). After incorporating the additional 2PACz molecules, the BE positions of the Pb 4f peaks in the spectra of the saturated PbS‐2PACz sample shifted toward higher BEs, which correlated with a notable increase in the intensity of the Pb─O coordination peaks, whereas a concurrent decrease in the intensity of the Pb–S coordination peaks was observed. Moreover, the P/Pb ratio of the PbS‐2PACz films was analyzed before and after introducing the additional 2PACz molecules, revealing an increase from 0.3 to 1.1 (Figure S6, Supporting Information). This trend provides strong evidence of increased 2PACz coverage and enhanced electrostatic interactions between the PbS surface and the phosphonic acid groups.

The effect of the additional 2PACz molecules on the bonding structure of the PbS‐2PACz film was assessed through grazing‐incidence wide‐angle X‐ray diffraction (GIWAXS) (Figure 3c). The 2PACz concentration in the IPA solution was systematically varied from 0 to 20 mm. The saturated PbS‐2PACz film formed from the solution with an additional 5 mm 2PACz retained its structure without any notable changes. In contrast, for the films formed with the 10 mm 2PACz solution, Bragg peaks emerged in the *q*_z_ (0.35 and 1.05 Å^−1^) and *q*_xy_ (1.46 and 1.58 Å^−1^) directions, indicating pronounced crystallinity. At higher concentrations (20 mm 2PACz), additional Bragg peaks were observed in the *q*_z_ (0.35, 0.73, and 1.05 Å^−1^) and *q*_xy_ (0.98, 1.07, 1.46, and 1.58 Å^−1^) directions, suggesting a greater degree of molecular ordering. These results suggest that 5 mm 2PACz is optimal for maintaining the structural integrity of the film, whereas excess 2PACz in the solution (10 mm and 20 mm) led to the formation of additional 2PACz layers on top of the film rather than further integration. To further examine whether the structural integrity of the film was maintained after incorporating the additional 2PACz, the sub‐monolayer PbS‐2PACz and saturated PbS‐2PACz were analyzed using FT‐IR spectroscopy and water‐contact angle measurements (Figure S7 and S8, Supporting Information). The absence of significant spectral shifts confirmed that the newly integrated 2PACz molecules maintained their assembled structures without undesirable secondary interactions. Consistently, the water contact angle of the saturated PbS–2PACz film (73°) showed no notable change, further indicating that the overall surface characteristics of the film remained unaltered.

The effectiveness of the incorporation of this extra 2PACz molecule in reducing under‐coordinated Pb defects was assessed using ss‐PL measurements (Figure 3d). Devices with glass/submonolayer PbS‐2PACz and glass/saturated PbS‐2PACz structures were prepared for comparison. For both films, a PL emission peak appeared at 1005 nm, indicating no change in the optical bandgap. However, the PL intensity of saturated PbS‐2PACz was five‐fold higher than that of sub‐monolayer PbS‐2PACz. This significant enhancement confirms that the trap states on the PbS surface were effectively passivated, thereby reducing non‐radiative recombination.

The surface passivation of PbS (111) with the 2PACz ligands was investigated using DFT calculations. Each Pb atom exposed on the surface of PbS (111) has one dangling electron (DE)^[^ ^40^ ^]^ (Figure S9, Supporting Information); thus, a single 2PACz ligand with a phosphonic anchoring group can passivate two surface Pb atoms. The calculations demonstrated that substituting two carboxylate ligands with one 2PACz ligand is thermodynamically favorable (Figure S10, Supporting Information), supporting the experimental ligand exchange process. Figure S11 (Supporting Information) shows that the single 2PACz per p(2 × 2) cell partially passivates the DEs of PbS (111), whereas the double 2PACz per p(2 × 2) cell achieve full passivation; in both cases, optical transitions from the carbazole‐localized in‐gap states to the conduction band are calculated to be effectively small as confirmed by their near‐zero transition dipole moments. The stabilization energy associated with coverage of the PbS (111) surface by 2PACz was also examined (Figure 3e). The partial passivation with single 2PACz ligand yielded a stabilization energy of 2.96 eV per ligand, whereas full passivation with double ligands enhanced the stabilization energy to 3.29 eV per ligand. This increase in the stabilization energy, rather than hindrance due to coulombic repulsion, as typically observed at higher ligand densities, is attributed to the favorable *π*–*π* stacking interactions between adjacent carbazole groups. Additionally, the analysis revealed that a higher ligand density induced a stronger dipole moment, which in turn led to a downward shift in the energy levels (Figure S12, Supporting Information). Taken together, these results highlight the effect of the 2PACz ligands in enhancing binding through close‐packed *π*–*π* stacking and precise control of the energy levels via dipole tuning, offering a dual advantage for efficient and tunable passivation of the PbS (111) surface.

To experimentally validate these effects, the band structures of saturated PbS‐2PACz and the perovskite layer were investigated using APS. As shown in Figure S13 (Supporting Information), the offset photon energy shifted from −5.47 eV for sub‐monolayer PbS‐2PACz to −5.60 eV for saturated PbS‐2PACz, aligning more favorably with the energy of the perovskite absorber layer (−5.65 eV). The corresponding energy‐level diagrams based on these modified band structures are shown in Figure 3f. As expected, this deeper VBM minimized the energy loss and facilitated efficient hole extraction, thereby contributing to increasing the *V_oc_*.^[^ ^19^ ^]^

### 2.4 Performance Enhancement in NBG PSCs and APTSCs via Saturated PbS‐2PACz HTL

**Figure 4:**
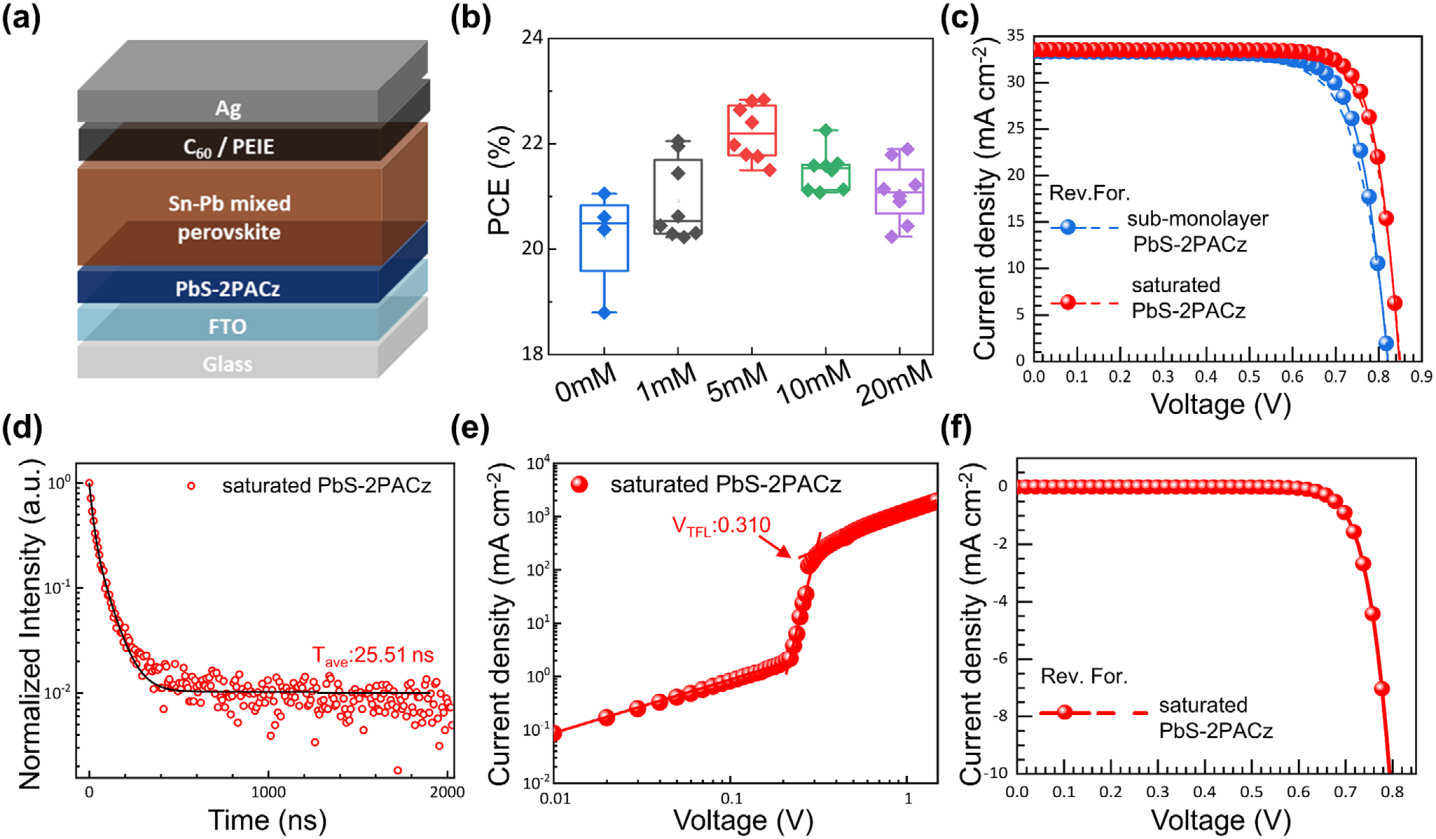
a) Schematic of NBG device structure. b) Statistical distribution of PCE parameters for NBG PSCs with varying 2PACz concentrations (0‒20 mm) c) *J–V* curves of NBG PSCs with sub‐monolayer PbS‐2PACz and saturated PbS‐2PACz as HTL. d) TR‐PL decay of NBG PSC films with saturated PbS‐2PACz (fitted to bi‐exponential function) confirming improved carrier dynamics. e) SCLC data for saturated PbS‐2PACz devices. Saturated PbS‐2PACz exhibits the lowest *V*_TFL_. f) Dark‐current characteristics of NBG PSCs with saturated PbS‐2PACz.

**Figure** **4**a shows the device architecture for the NBG PSCs with a glass/FTO/HTL/NBG perovskite/fullerene‐C_60_ (C_60_)/polyethylenimine (PEIE)/Ag configuration. To optimize the ligand density on the surface, the concentration of the 2PACz solution was systematically varied from 1  to 20 mm. From the box‐plots of the photovoltaic parameters (Figure 4b), the optimal concentration was determined to be 5 mm, which yielded saturated PbS‐2PACz films with the highest average PCE and the narrowest distribution (PCE: 22.22 ± 0.55%, *J_sc_* = 33.50 mA cm^−2^, *V_oc_* = 0.850 V, FF = 80.24%). Figure 4c presents a comparison of the *J–V* characteristics of the best‐performing devices employing sub‐monolayer and saturated PbS‐2PACz. Notably, compared with the devices employing sub‐monolayer PbS‐2PACz (Figure S14, Supporting Information), the devices with saturated PbS‐2PACz had a significantly higher *V_oc_* and FF, attributed to the improved energy‐level alignment and enhanced interfacial passivation. The photovoltaic parameters are listed in **Table** **1**. Furthermore, the integrated *J_sc_* value (33.12 mA cm^−2^) obtained from the external quantum efficiency (EQE) spectra was in close agreement with the *J–V* results (Figure S15, Supporting Information), confirming the reliability of the measurements. In contrast, the *V_oc_*, FF, and overall PCE were lower for the devices employing oversaturated PbS‐2PACz (with 10  or 20 mm 2PACz solutions) (Figure S16, Supporting Information). This decline is likely due to the formation of excess 2PACz aggregates or self‐assembled multilayers, which can disrupt the interfacial contact and introduce energetic disorder, thereby impeding efficient charge extraction.

**Table 1:** Photovoltaic performance metrics of NBG PSCs with sub‐monolayer PbS‐2PACz and saturated PbS‐2PACz.

HTL	Bias	*V_oc_* [V]	*J_sc_* [mA cm^−2^]	FF [%]	PCE [%]	Integr‐ated *J_sc_*
sub‐monolayer PbS‐PACz	Champion	0.823	33.29	76.85	21.05	33.09
	Average	0.782 ± 0.05	32.63 ± 0.53	70.67 ± 5.64	18.13 ± 2.55	
saturated PbS‐PACz	Champion	0.850	33.50	80.24	22.84	33.12
	Average	0.844 ± 0.01	33.20 ± 0.33	79.29 ± 0.70	22.22 ± 0.55	

TR‐PL was used to evaluate the hole‐extraction properties of saturated PbS‐2PACz (Figure 4d), and the results are listed in Table S4 (Supporting Information). Saturated PbS‐2PACz exhibited the shortest decay lifetimes for the fast (τ_1_: 13.13 ns) and slow (τ_2_: 51.60 ns) components, with the fast decay fraction becoming dominant (A_1_: 0.68, and A_2_: 0.32). Consequently, PbS‐2PACz afforded the lowest τ_avg_ of 25.51 ns, reflecting efficient suppression of non‐radiative recombination and rapid charge transfer. To further investigate the charge transport characteristics, SCLC measurements were also performed under identical conditions using three independently fabricated hole‐only devices (Figure 4e; Figure S17, Supporting Information). The device incorporating saturated PbS‐2PACz exhibited the lowest *V*_TFL_ (0.310 V) and *N*_trap_ (9.66 ± 0.20 × 10^14^ cm^−3^), confirming a reduction in the trap‐assisted recombination. Moreover, dark *J*–*V* measurements revealed that PbS‐2PACz afforded the highest offset voltage (0.64 V), further indicating enhanced charge‐transport properties. To determine whether saturated PbS‐2PACz affected the crystallinity of the NBG perovskite, GIWAXS analyses were conducted, and top‐ and side‐view SEM images were acquired (Figures S18 and S19, Supporting Information). No significant changes in the grain structure or crystallinity of the NBG perovskite were observed, confirming that the introduction of additional 2PACz molecules primarily enhanced the interfacial passivation rather than altering the crystallization of the NBG perovskite. These findings indicate that the improved device performance is not attributable to enhanced crystallinity of the perovskite, but rather to the optimized interfacial charge dynamics, including more efficient hole extraction and reduced defect‐induced recombination, facilitated by saturated PbS‐2PACz. To further evaluate the versatility of PbS–2PACz as a hole transport layer beyond narrow‐bandgap applications, saturated PbS–2PACz was implemented in a wide‐bandgap perovskite system (Cs_0_._2_FA_0_._55_MA_0_._25_Pb(I_0_._85_Br_0_._15_)_3_; bandgap: 1.71 eV). As shown in Figure S20 and Table S5 (Supporting Information), the resulting device exhibited stable photovoltaic performance (PCE: 19.13%, *J*_sc_ = 19.84 mA cm^−2^, *V*_oc_ = 1.265 V, FF = 76.21%), confirming the compatibility of PbS–2PACz with a broader range of perovskite compositions. These findings demonstrate that PbS–2PACz serves as a versatile and broadly applicable hole transport layer across diverse perovskite bandgaps.

**Figure 5:**
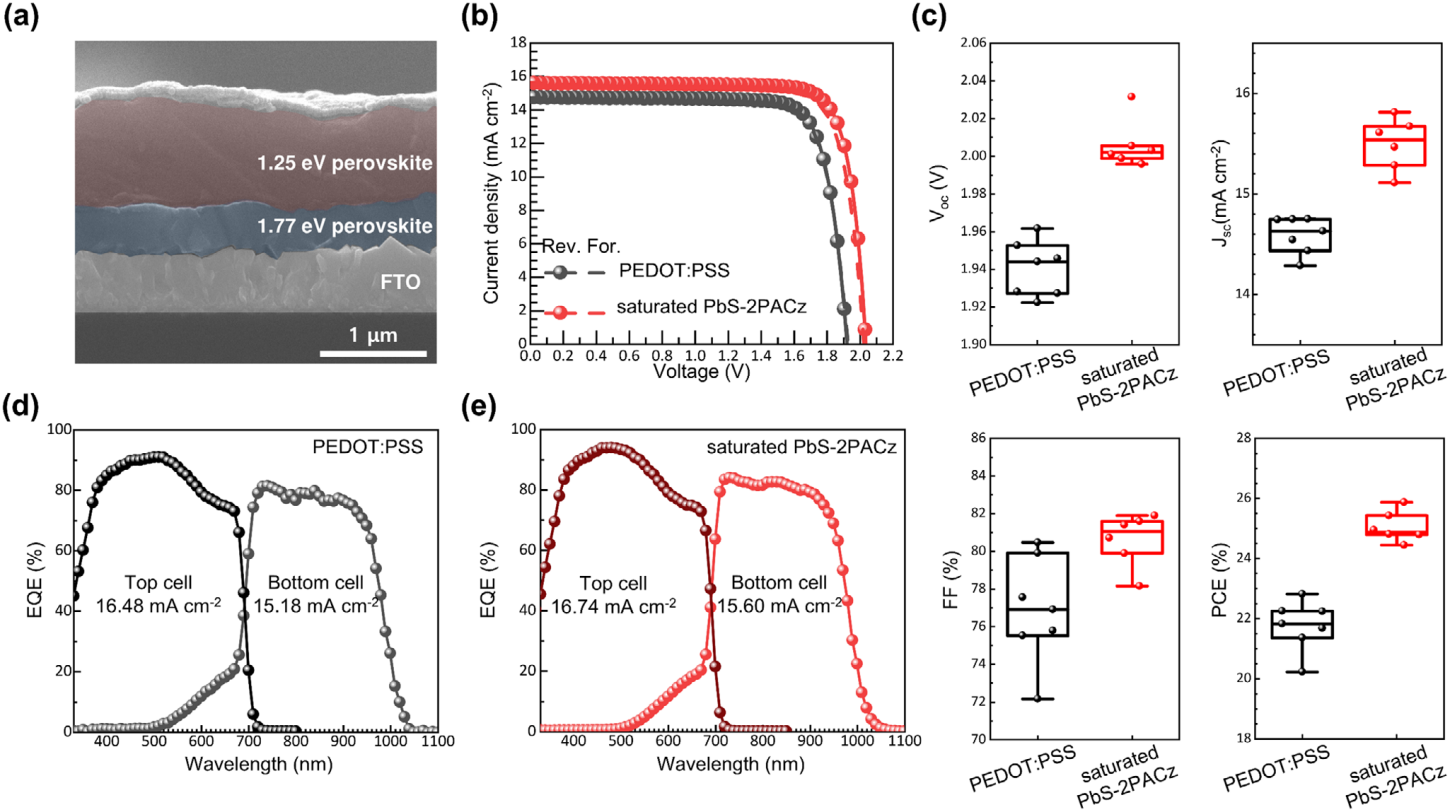
a) Cross sectional SEM image of all‐perovskite two‐terminal tandem solar cell, showcasing the layered architecture and uniform thicknesses of the top and bottom absorber layers. b) *J–V* curves of champion tandem devices fabricated with PEDOT:PSS and saturated PbS‐2PACz as HTL, demonstrating enhanced photovoltaic performance with optimized HTL. c) Box plots comparing key photovoltaic parameters for tandem devices utilizing PEDOT:PSS versus saturated PbS‐2PACz, illustrating improvements in efficiency. EQE spectra for tandem cells based on d) PEDOT:PSS and e) saturated PbS‐2PACz.

To evaluate the effectiveness, saturated PbS‐2PACz was used to fabricate tandem devices, where a 1.77 eV WBG PSC/FA_0.6_MA_0.4_Pb(I_0.6_Br_0.4_)_3_‐based front subcell was employed with a typical PCE of 18.17%, *V_oc_* of 1.246 V, *J_sc_* of 17.53 mA cm^−2^, and FF of 83.20% (more details are provided in Figure S21 and Table S6, Supporting Information). The cross sectional SEM image of the tandem device (**Figure** **5**a) illustrates the architecture comprising glass/FTO/HTL/WBG perovskite/ETL/interconnecting layer (ICL)/saturated PbS‐2PACz/NBG perovskite/C_60_/PEIE /Ag. Figure 5b shows the *J*–*V* curves of the best‐performing tandem devices employing either PEDOT:PSS or saturated PbS‐2PACz as the HTL. The tandem device employing saturated PbS‐2PACz achieved a PCE of 25.95% (*J_sc_* = 15.61 mA cm^−2^, *V* ***_oc_*** = 2.035 V, FF = 81.67%), outperforming the PEDOT:PSS‐based tandem device, for which the PCE was 22.82% (*J* ***_sc_*** = 14.75 mA cm^−2^, *V* ***_oc_*** = 1.922 V, FF = 80.47%). The detailed photovoltaic parameters are listed in **Table** **2**. The box‐charts of the photovoltaic parameters show that the reproducibility of the tandem devices employing saturated PbS‐2PACz is superior to that of the devices with PEDOT:PSS, as evidenced by the narrower PCE distribution (PCE: 25.05% ± 0.41) of the former. The *J_sc_* values derived from the *J*–*V* curves matched well with the EQE spectra of the front and back subcells (Figure 5d,e). This study demonstrates that the saturated PbS‐2PACz film is a highly effective HTL for APTSCs.

**Table 2:** Photovoltaic performance metrics of APTSCs with PEDOT:PSS and saturated PbS‐2PACz.

HTL	Bias	*V_oc_* [V]	*J_sc_* [mA cm^−2^]	FF [%]	PCE [%]
PEDOT:PSS	Champion	1.922	14.75	80.47	22.82
	Average	1.939 ± 0.014	14.59 ± 0.17	76.37 ± 3.02	21.60 ± 0.91
Saturated PbS‐2PACz	Champion	2.035	15.61	81.67	25.95
	Average	2.006 ± 0.014	15.49 ± 0.16	80.61 ± 0.48	25.05 ± 0.41

## 3 Conclusion

PbS‐2PACz was introduced as a novel HTL to address the key limitations of the interface in NBG PSCs. Unlike conventional 2PACz, which suffers from poor solubility, micelle aggregation, and weak adhesion to metal oxides, the PbS‐2PACz film enables stronger interfacial bonding, improved film uniformity, and enhanced charge transport. These features were achieved by leveraging a multiscale assembly strategy, in which 2PACz molecules were chemically anchored onto the surfaces of PbS before film deposition, forming a stable and well‐ordered interfacial structure. This strategy mitigates micelle‐induced defects observed in conventional 2PACz‐based HTLs and ensures the formation of a uniform monolayer without pinholes. To further optimize the interfacial passivation and energy‐level alignment, additional 2PACz molecules were incorporated into the PbS‐2PACz film via spin coating. This additional 2PACz integration resulted in a denser and more compact molecular arrangement, which significantly improved trap passivation and reduced interfacial recombination. These modifications translated into substantial enhancements in the device performance, with single‐junction NBG PSCs achieving a remarkable PCE of 22.84% and APTSCs employing saturated PbS‐2PACz achieving a PCE of 25.95%. Beyond these immediate gains in the single‐ and tandem junction performance, this work underscores the critical role of interfacial engineering in reducing recombination losses and optimizing charge transport. Furthermore, the methodologies presented herein offer a versatile framework for the advancement of solution‐processed perovskite electronics. By fine‐tuning the passivation strategies, this approach paves the way for large‐area, stable, and commercially viable perovskite solar modules.

## 4 Experimental Section

### Materials

All materials were used as received without further purification. Lead(II) acetate trihydrate (Pb(Ac)_2_·3H_2_O, 99.999%), oleic acid (OA, 99%), 1‐octadecene (ODE, 90%), toluene (99.5%), acetone (99.5%), octane (95%), zinc acetate dehydrate (>98%), potassium hydroxide (KOH, >95%), methanol (99.5%), ethanol (95%), methylammonium iodide (MAI, >99.0%), formamidinium iodide (FAI, >99.0%), and methylammonium chloride (MACl, >99.99%) were purchased from GreatCell Solar Materials. Tin(II) iodide (SnI_2_, beads, >99.99% trace metals basis), tin(II) fluoride (SnF_2_, >99.0%), ethylenediamine (EDA, >99%), dimethyl sulfoxide (DMSO, 99.9% anhydrous), *N,N*‐dimethylformamide (DMF, 99.8% anhydrous), 2‐propanol (IPA, 99.5% anhydrous), toluene (Tol, 99.8% anhydrous), chlorobenzene (CB, 99.8% anhydrous), 1,2‐difluorobenzene (DFB2, 98%), methanol (MeOH, 99.8% anhydrous), and polyethyleneimine 80% ethoxylated solution (PEIE, 37 wt.% in H_2_O) were purchased from Sigma‐Aldrich. Bis(trimethylsilyl) sulfide ((TMS)_2_S, 99.9%) was purchased from JSI Silicon. Ammonium iodide (≥98%), lead(II) iodide (PbI_2_, 99.99%, trace metal basis), and 2PACz (>98.0%) were purchased from TCI. Fullerene C_60_ was purchased from 1‐Material. An aqueous solution of poly[3, 4‐ethylenedioxythiophene]:poly(styrene sulfonate) (PEDOT:PSS; Clevios PVP AI 4083) was purchased from Heraeus.

### 1.25 eV NBG FA_0.7_MA_0.3_Pb_0.5_Sn_0.5_I_3_ Perovskite

The precursor solution was prepared in a mixed solvent system of DMF and DMSO in a 2:1 volume ratio. The molar ratios of FAI/MAI and PbI_2_/SnI_2_ were set to 0.7:0.3 and 0.5:0.5, respectively. The total precursor concentration was set to 1.8 m. SnF_2_ was added at a concentration of 10 mol% relative to SnI_2_. The solution was stirred at room temperature for 2 h and subsequently filtered through a 0.20 µm polytetrafluoroethylene (PTFE) filter prior to depositing the perovskite film.

### 1.71 eV WBG Cs_0.2_ FA_0.55_MA_0.25_Pb(I_0.85_Br_0.15_)_3_ Perovskite

The precursor solution was prepared in a mixed solvent system of DMF and DMSO with a 4:1 volume ratio. The molar ratios of CsI/FAI/MAI/MABr and PbI_2_/PbBr_2_ were set to 0.2:0.55:0.1:0.15 and 0.85:0.15, respectively. The total precursor concentration was set at 1.2 m. The 10 mol% of MAPbCl_3_ was added to the precursor solution. The solution was stirred at room temperature overnight and subsequently filtered through a 0.20 µm polytetrafluoroethylene (PTFE) filter prior to depositing the perovskite film.

### 1.80 eV WBG FA_0.6_MA_0.4_Pb(I_0.6_Br_0.4_)_3_ Perovskite

The precursor solution was prepared in a mixed solvent system of DMF and DMSO with a 4:1 volume ratio. The molar ratios of FAI/MAI and PbI_2_/PbBr_2_ were set to 0.6:0.4 and 0.6:0.4, respectively. The total precursor concentration was set at 1.15 m. Excess PbI_2_ (6 mol%) was added to the precursor solution. The solution was stirred at room temperature overnight and subsequently filtered through a 0.20 µm polytetrafluoroethylene (PTFE) filter prior to depositing the perovskite film.

### Synthesis of PbS‐OA CQDs

Lead(II) acetate trihydrate (Sigma–Aldrich, 99.999%), OA (Alfa Aesar,99%), 1‐octadecence (Sigma–Aldrich, 90%), and bis(trimethylsilyl) sulfide (Sigma–Aldrich) were used as purchased without further purification. The OA‐capped PbS CQDs were prepared as previously described.^[^ ^44^ ^]^

### Purification of PbS‐OA CQDs by Precipitation‐Redispersion

The first precipitation was conducted by injecting acetone into the crude solution. After the first precipitate was dispersed in toluene, the second precipitate was obtained by injecting a mixture of acetone and methanol into the solution of PbS CQDs in toluene. The final precipitation was conducted by injecting methanol into the solution of PbS CQDs in toluene. Anhydrous solvents were used, and purification was performed in a nitrogen‐filled glove box.

### Precipitation of PbS‐2PACz

2PACz (0.3 m) was dissolved in DMF (3 mL) and CB (1 mL) and added to the PbS‐OA solution (6 mL of 30 mg mL^−1^ in octane) and vortexed for 10 min until the PbS CQDs were transferred to the DMF and CB co‐solvent phase. The transferred CQDs in the DMF and CB cosolvents were then washed three times with octane. Subsequently, the precipitated CQDs were dispersed in 2 mL of CB, and 6 mL of octane was added to precipitate the CQDs. Finally, the precipitated CQDs were dried under vacuum and redispersed in the desired solvent at the desired concentration.

### Sn‐Pb Mixed Perovskite Solar Cells

Patterned fluorine‐doped tin oxide (FTO, 8 Ω sq^−1^, Tec 8, Pilkington, United Kingdom) substrates (2.5 × 2.5 cm^2^) were sequentially cleaned with deionized water, acetone, and IPA, dried with a N_2_ air gun, and treated with ozone plasma. After cleaning, the substrates were immediately transferred to a N_2_‐filled glove box (H_2_O, O_2_ < 0.3 ppm) to deposit the HTL and perovskite films. The PbS‐2PACz solution with CB (80 µL) was spin‐coated onto the FTO substrates at 3,000 rpm for 30 s (sub‐monolayer PbS‐2PACz). To form saturated PbS‐2PACz, a solution of 2PACz in IPA (100 µL) was spin‐coated onto the sub‐monolayer PbS‐2PACz at 3,000 rpm for 30 s, followed by annealing on a hotplate at 100 °C for 10 min. After cooling, the film was rinsed with IPA (100 µL) under the same spin‐coating conditions used for PbS‐2PACz deposition. The perovskite films were deposited using a two‐step spin‐coating process with the following parameters: 1) 1,000 rpm for 10 s with an acceleration of 200 rpm s^−1^ and 2) 4,000 rpm for 40 s with a ramp rate of 1,000 rpm s^−1^. The CB/DFB2 mixed solution (400 µL) was dropped onto the spinning substrate 20 s before the end of the second spin‐coating step. The substrates were then annealed at 100 °C for 10 min on a hotplate. For post‐treatment with ethylenediamine (EDA), 6.01 mg of EDA was dissolved in 1,000 mL of toluene. The solution was stirred at 25 °C for 3 h and subsequently filtered through a 0.20 µm PTFE filter before spin‐coating. The prepared EDA solution (40 µL) was spin‐coated onto the perovskite films at 5000 rpm for 50 s, followed by annealing at 75 °C for 5 min. A 35 nm‐thick C_60_ layer was deposited via thermal evaporation at a rate of 0.1 Å s^−1^. After depositing C_60_, a 0.2 wt.% PEIE solution in MeOH (100 µL) was spin‐coated onto the assembly at 4,000 rpm for 30 s, followed by annealing at 100 °C for 1 min. The top electrode was formed by depositing 100 nm of silver by thermal evaporation using a shadow mask. The deposition rate was set to 0.1 Å s^−1^ until the film thickness reached 20 nm, then increased to 1 Å s^−1^ to achieve the target thickness.

### 1.71 eV WBG Perovskite Solar Cells

The processes from TCO preparation to PbS‐2PACz HTL formation were the same as those applied to the Sn‐Pb perovskite solar cells, with indium tin oxide (ITO, 15 Ω sq^−1^) substrates substituted for FTO. The prepared 1.71 eV WBG perovskite precursor was deposited on the PbS‐2PACz layer using a two‐step spin‐coating process with the following parameters: 1) 2,000 rpm for 3 s with an a ramp rate of 1,000 rpm s^−1^ and 2) 4,000 rpm for 40 s with a ramp rate of 2,000 rpm s^−1^. The ethyl acetate (EA) solution (300 µL) was dropped onto the spinning substrate 14 s before the end of the second spin‐coating step. The substrates were then annealed at 120 °C for 10 min on a hotplate. The processes from post‐treatment of perovskite to top silver electrode deposition were the same as those applied to the Sn‐Pb perovskite solar cells

### All‐Perovskite Tandem Solar Cells

Patterned FTO substrates were prepared by following the same cleaning process described above. 2PACz solution (1 mm) was prepared by dissolving 2PACz powder in IPA. This solution was spin‐coated onto an FTO substrate at 3,000 rpm for 30 s, followed by annealing at 100 °C for 10 min. The WBG perovskite precursor solution (100 µL) was spin‐coated onto the substrates at 6,000 rpm for 30 s. CB (200 µL) was dropped onto the substrate 25 s before the end of the spin‐coating process. The substrates were then annealed at 100 °C for 20 min on a hotplate. After annealing the perovskite, 100 µL of EDA solution (0.1 mm EDA in toluene) was spin‐coated onto the perovskite films at 3000 rpm for 30 s, followed by annealing on a hotplate at 65 °C for 5 min. The substrates were cooled and then transferred to an evaporation chamber to deposit a 20 nm C_60_ layer. After depositing C_60_ on the WBG perovskite layer, the samples were transferred to an atomic layer deposition system (CN1, Atomic Classic). A 25 nm SnO_2_ layer was deposited using tetrakis(dimethylamino) tin(IV) precursor and deionized water at 90 °C. Subsequently, graphene (as a recombination layer) was deposited on the SnO_2_ layer under ambient conditions. The remaining fabrication steps were identical to those for the single‐junction NBG device.

### DFT Calculation

First‐principles density functional theory (DFT) calculations were performed using the Kohn–Sham formalism with projector augmented‐wave (PAW) potentials and the Perdew–Burke–Ernzerhof (PBE) exchange‐correlation functional, as implemented in the Vienna Ab initio Simulation Package (VASP). A plane‐wave kinetic energy cut‐off of 400 eV was employed. To account for the van der Waals interactions between the molecular complexes, a DFT‐D4 dispersion correction scheme was applied. The surface models were subjected to structural relaxation until the Hellmann–Feynman atomic forces reached < 0.02 eV Å^−1^. Brillouin zone sampling was conducted using a Γ‐centered 4 × 4 × 1 k‐point mesh. The calculated lattice constant of 5.993 Å for rock‐salt PbS is in good agreement with the experimental value of 5.936 Å. For the surface calculations, PbS (111) p(2 × 2) slab models were constructed with six atomic double layers (Figure S9, Supporting Information), with a vacuum region larger than 15 Å along the surface‐normal direction. The S‐terminated bottom surface was passivated by hydrogen atoms, and the bottom two atomic double layers (including the hydrogen atoms) were fixed during relaxation. A dipole correction was employed for the slab calculation. The stabilization energy per ligand is defined as:

(1) $$E_{\mathrm{stabilization}}=\left[E\left(\mathrm{surface}\right)+nE\left(LH_{2}\right)-nE\left(H_{2}\right)-E\left(nL/\mathrm{surface}\right)\right]\;/n$$

where *n* is the number of ligands, and LH_2_ represents the corresponding acid of the ligand.

### Characterization

Absorption spectra were acquired using an ultraviolet/visible/near‐infrared spectrophotometer (Shimadzu, UV3600). The photoluminescence (PL) spectra were obtained using a PL spectrometer (Fluorolog, Horiba Jobin Yvon). PbS‐OA and PbS‐2PACz were dissolved in chloroform for the absorption and PL measurements. XPS data were acquired using a PHI Quantera‐II spectrometer with an Al anode (Al Kα = 1486.7 eV). The samples for XPS analysis were spin‐coated onto Si wafers. Scanning electron microscopy (SEM) images were collected using a FE‐SEM (Hitachi S‐4800) instrument operated at an accelerating voltage of 2 kV. Dark *J–V* curves were acquired using a Keithley 2400 source meter. The configuration of the hole‐only devices for space‐charge‐limited current (SCLC) analysis was as follows: glass/FTO/HTL/NBG perovskite/Ag. Steady‐state photoluminescence (ss‐PL) and time‐resolved PL (TR‐PL) spectra were obtained using a Horiba DeltaDiode‐485L system. Steady‐state PL measurements were conducted using a 485 nm diode laser (Horiba, DeltaDiode‐485L) with a repetition rate of 100 MHz. TR‐PL data were acquired in the pulsed mode with a diode laser at 485 nm (Horiba, DeltaDiode‐485L‐CW). The emitted photons were collected using a double‐grating monochromator (Horiba, FL‐1005) and detected using a liquid nitrogen‐cooled, low‐noise photomultiplier tube (Hamamatsu, R5509‐43).

### Characterization of Solar Cells

The *J–V* characteristics of the cells were measured using a Keithley 2400 source meter under illumination with a solar simulator (Newport Oriel Sol3A) at an AM 1.5G light intensity of 100 mW cm^−2^ with a xenon lamp calibrated versus reference cells (KG‐5 and KG‐0 reference cells were used in analyzing the WBG and NBG PSCs, respectively). The *J–V* characteristics were analyzed in the forward and reverse bias directions over the voltage range of −0.1 to 0.9 V for the single‐junction NBG PSCs and −0.2 to 2.1 V for the all‐perovskite tandem solar cells, with a step size of 10 mV and a step delay of 30 ms. The active area was defined by aperture shade masks (0.0625 cm^2^). The EQE spectra of the single‐junction devices were acquired using a Newport Oriel IQE 200 system. To analyze the front and back subcells, bias illumination from high‐intensity LEDs with emission peaks at 850 and 450 nm was used for the all‐PRV tandem solar cells.

## Conflict of Interest

The authors declare no conflict of interest.

## Author Contributions

S.H.H. and S.L. contributed equally to this study. S.H.H. conceived the idea, synthesized and characterized the materials, and wrote the manuscript. S.L. conceived the idea, fabricated, and characterized the devices, and co‐wrote the manuscript. Sunwoo Kim fabricated the wide‐bandgap subcells for tandem device integration. J.J., D.S., and M.J. performed the DFT calculations. E.C., J.K., and M.K. conducted the XPS and GIWAXS measurements and contributed to data interpretation. M.C. performed APS analysis. T.G. analyzed the performance of the FET device. S.J.P., J.R.L., and G.C. contributed to the device fabrication and characterization. S.L. provided conceptual inputs and edited the manuscript. Y.‐H.K. advised on DFT analysis and provided guidance for data interpretation and manuscript writing. D.H.K. and S.J. supervised the project, provided guidance on data interpretation, and contributed to writing the manuscript.

## Supporting information



Supporting Information

## Data Availability

The data that support the findings of this study are available in the supplementary material of this article.
