# Supplementary material for: Chemically Anchored PbS‐2PACz CQDs Inks for Scalable HTL in Narrow‐Bandgap and All‐Perovskite Tandem Solar Cells
Source: Small. 2025 Sep 27;21(46):e05059. doi: 10.1002/smll.202505059 (PMC12632428; doi:10.1002/smll.202505059)
Supplement: Supplementary file 1 — Supporting Information [file SMLL-21-e05059-s001.docx]

Supporting Information

Chemically Anchored PbS-2PACz CQDs Inks for Scalable HTL in Narrow-Bandgap and All-Perovskite Tandem Solar Cells

Seung Hwa Hong,^a,1^ Sangheon Lee,^b,1^ Sunwoo Kim,^c^ Jaegwan Jung,^d^ Doeun Shim,^a^ Eunhye Cho,^a^ Minwoo Jeong,^e^ Mahnmin Choi,^a^ Taewon Goo,^a^ Jugyoung Kim,^a^ Sang Jun Park,^b^ Meeree Kim,^a^  Jae Ryoung Lee,^b^ Gabeen Cho,^b^ Sangwook Lee,^c,^* Yong-Hyun Kim,^d,e,f,^* Dong Hoe Kim^b,^* and Sohee Jeong^a,g,h,^*


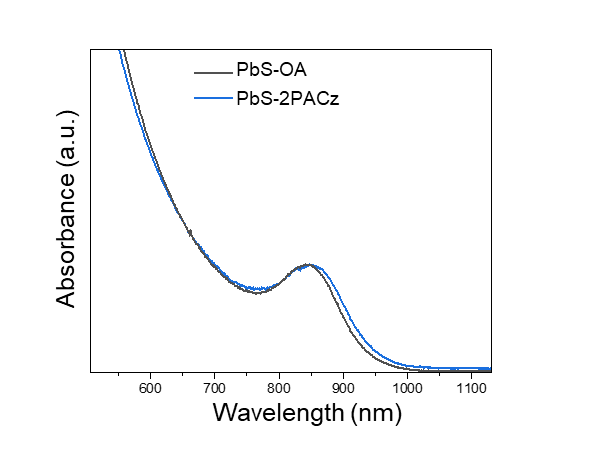


**Figure S1.** UV-vis absorption spectra of PbS-OA and PbS-2PACz in chloroform.


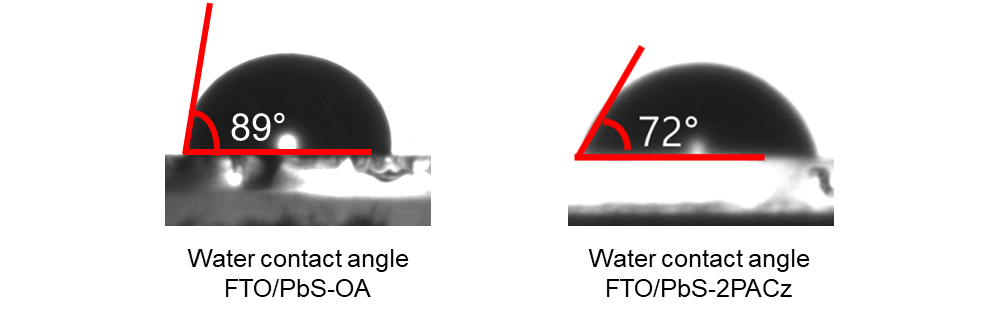


**Figure S2.** Water contact angle measurements for FTO/PbS-OA and FTO/PbS-2PACz surface demonstrating its surface wettability and confirming binding of phosphonic acid groups to PbS surface.


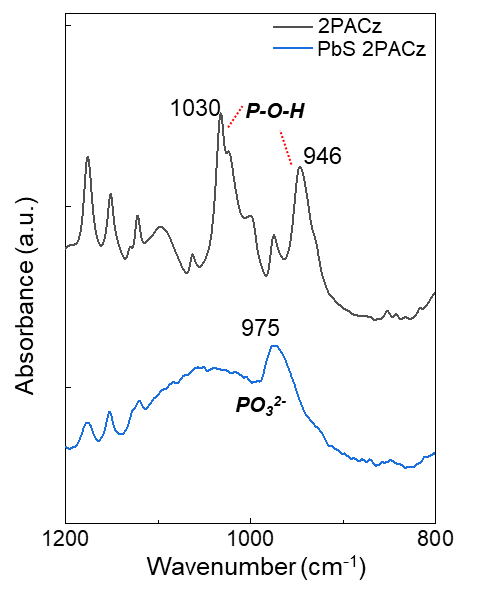


**Figure S3.** FT-IR spectra 2PACz and PbS-2PACz, confirming 2PACz ligand bind in in bi/tridentate coordination to the surface Pb.


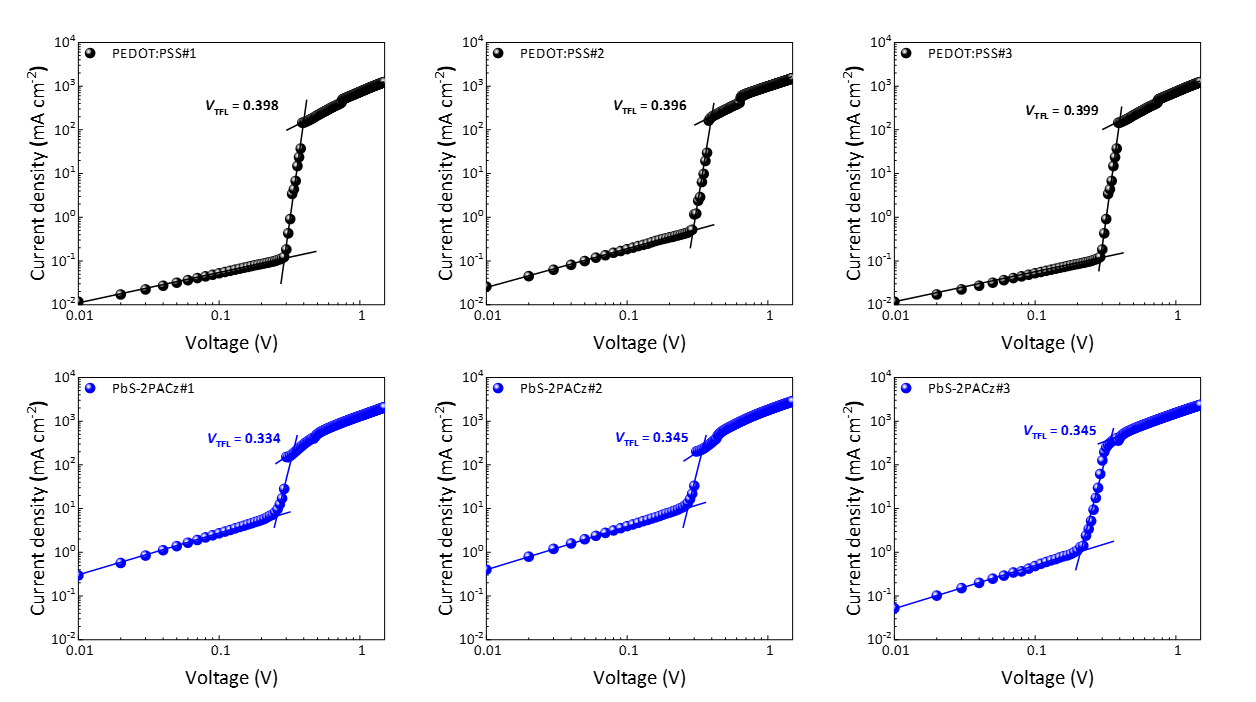


**Figure S4.** SCLC measurements for PEDOT:PSS and PbS-2PACz hole-only devices.


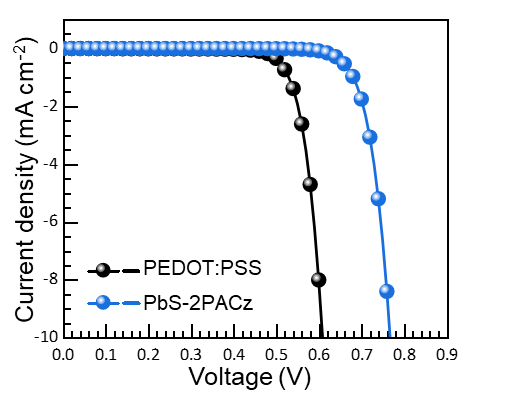


**Figure S5.** Dark-current characteristics of NBG PSCs utilizing PEDOT:PSS and PbS-2PACz as HTL.

**
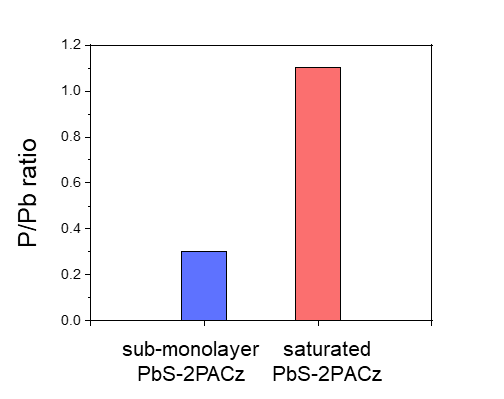
**

**Figure S6.** Comparison of P/Pb atomic ratio of sub-monolayer PbS-2PACz and saturated PbS-2PACz, showing increased 2PACz incorporation.


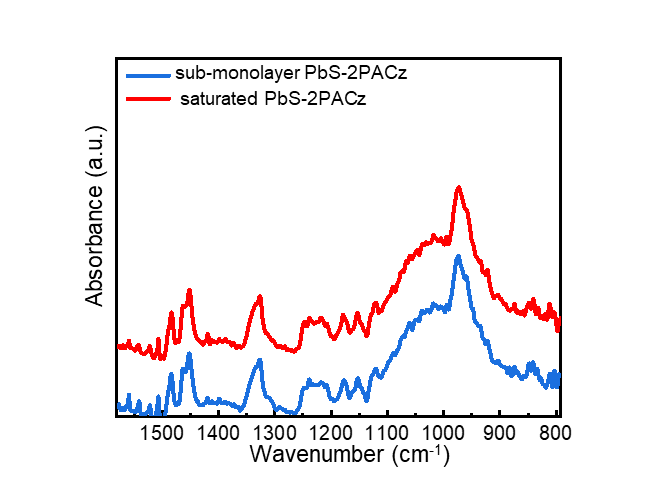


**Figure S7.** FT-IR spectra of sub-monolayer PbS-2PACz (blue) and saturated PbS-2PACz (red). The absence of significant changes in the FT-IR spectra suggests that the additional 2PACz solution did not alter the surface-binding mode of the phosphonic groups on the PbS CQDs.


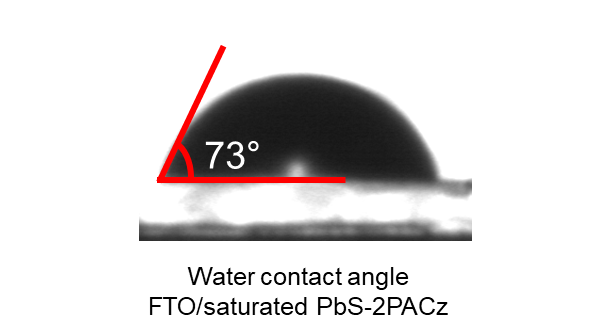
**Figure S8.** Water contact angle measurement of the FTO/saturated PbS-2PACz surface.


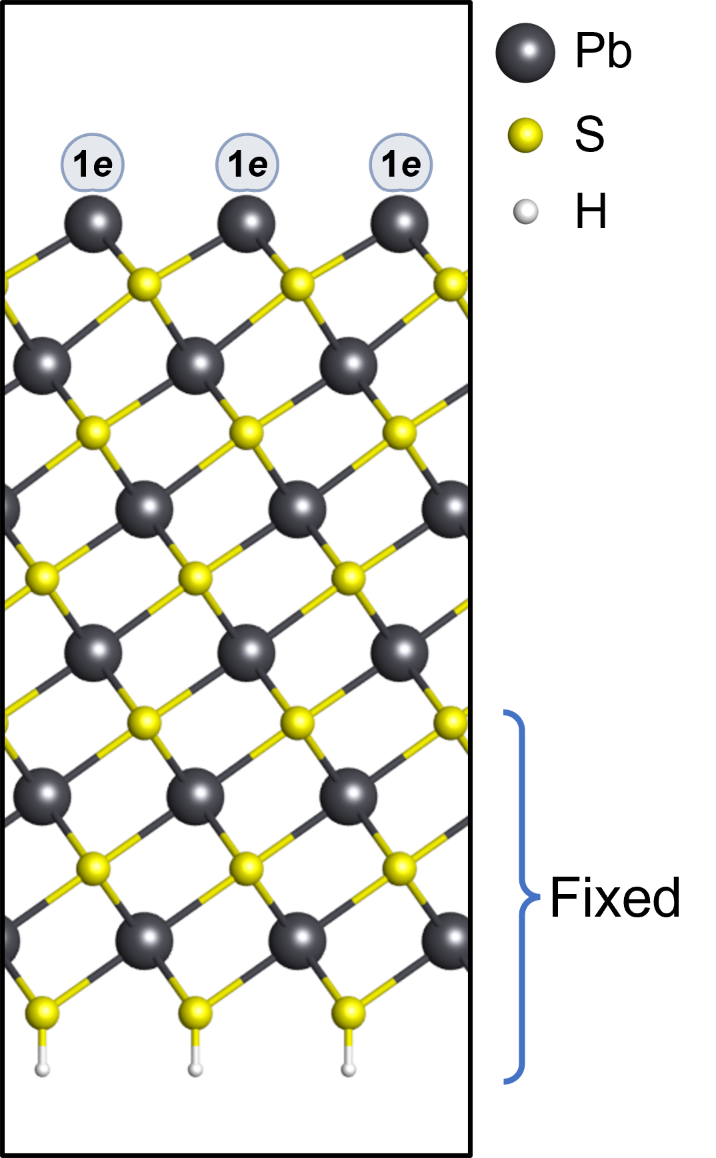


**Figure S9.** Slab model of the atomic structure of bare PbS (111). The bottom-surface S atoms were passivated by hydrogen atoms.


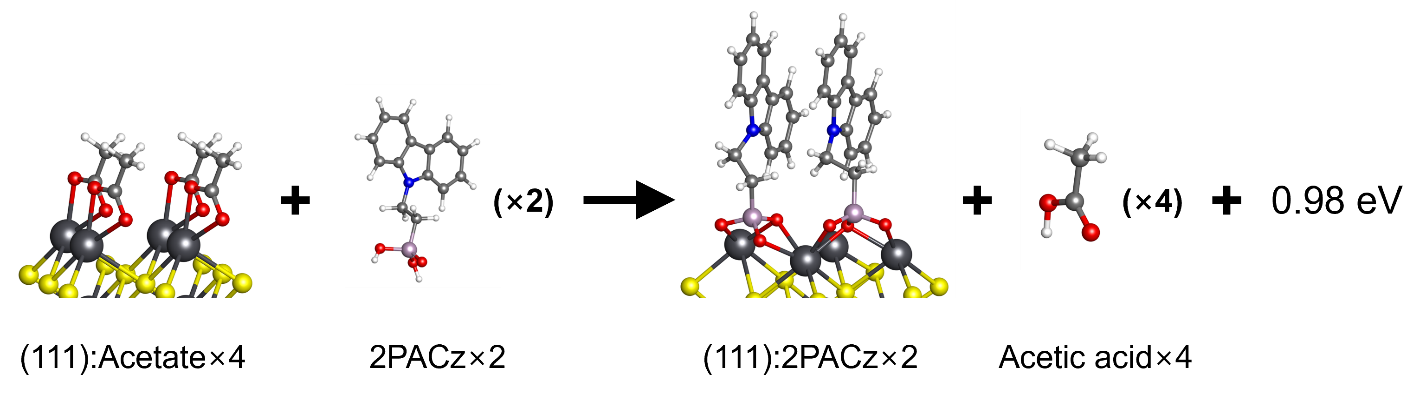


**Figure S10.** Calculated chemical reaction involving ligand exchange from carboxylate acid to 2PACz. On the PbS (111) p(2 × 2) surface, substituting four acetate ligands with two 2PACz yields an energy benefit of 0.98 eV, indicating favorable ligand exchange.


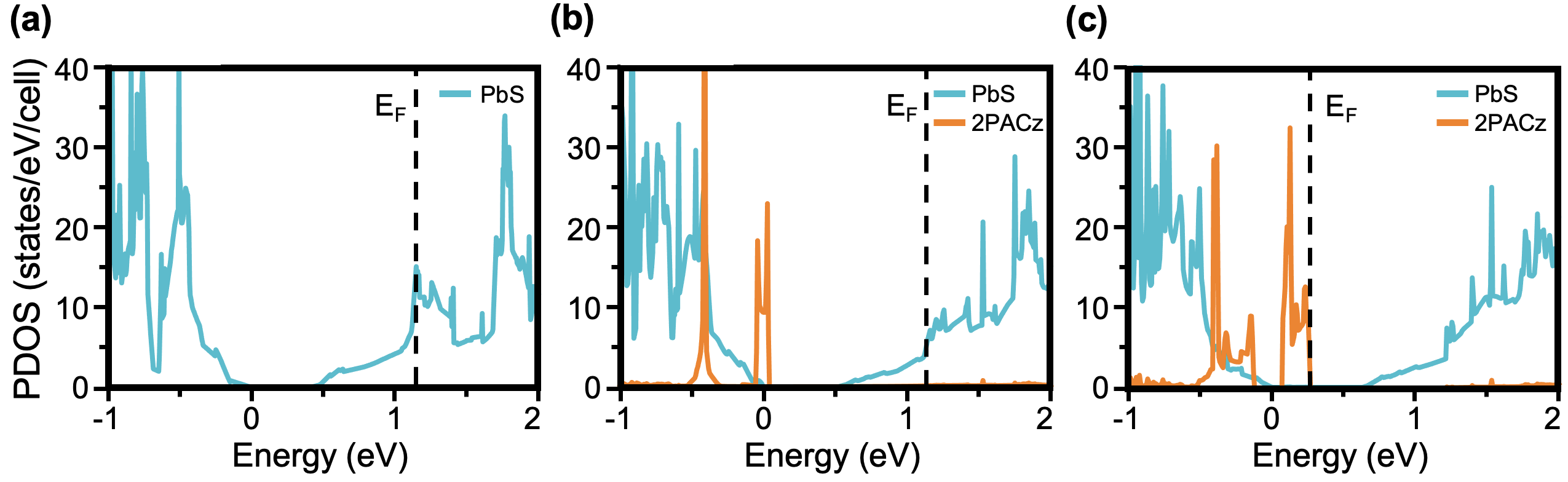


**Figure S11.** Projected density of states (PDOS) plots of PbS (111) p(2 × 2) slab with passivating 2PACz ligands: (a) no 2PACz, (b) single 2PACz, and (c) two 2PACz molecules per cell. The bulk valence band maximum (VBM) of PbS was aligned to 0 eV; Fermi level (*E*_F_) is indicated by dashed line. While dangling electrons remain in (a) and (b), they are completely passivated in (c). In cases (b) and (c), optical transitions from the carbazole-localized in-gap states to the conduction band are forbidden, as confirmed by their near-zero transition dipole moments.


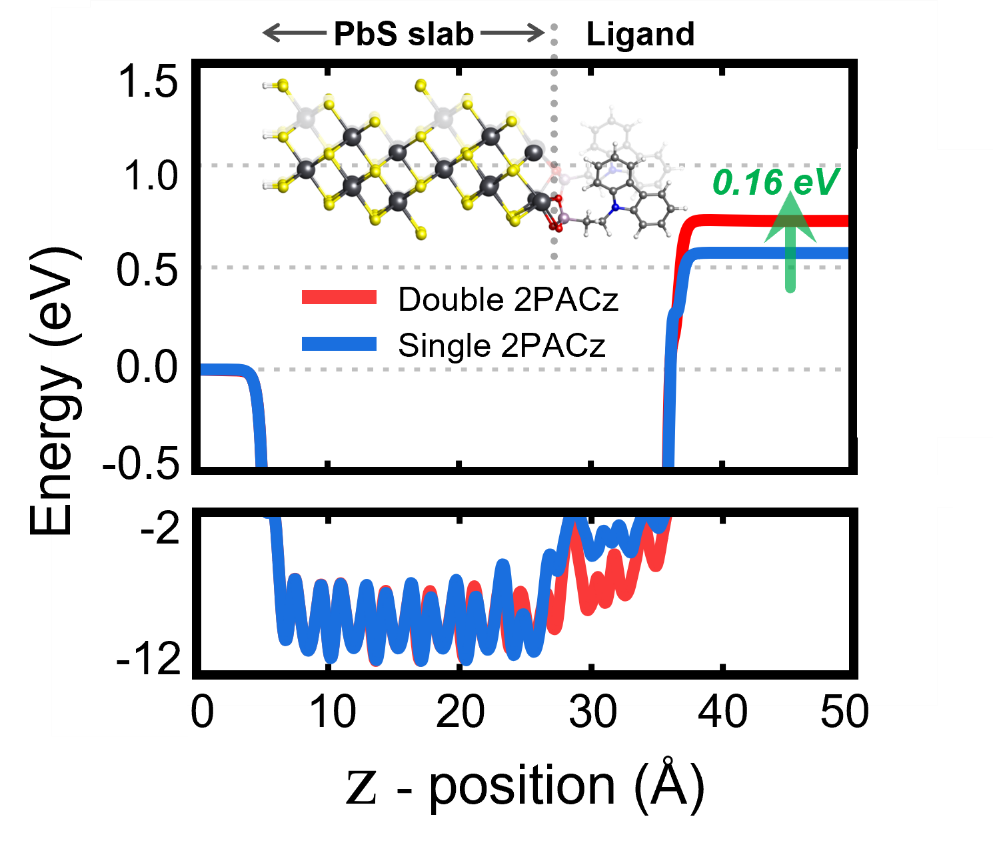


**Figure S12.** Calculated energy-level shifts induced by varying 2PACz coverage on p(2 × 2) PbS (111) surface. Complete passivation with double 2PACz ligands raises the vacuum level by 0.16 eV compared with the partially passivated single-ligand case, consistent with the experimentally observed lowering of the energy levels in **Figure 3f**.


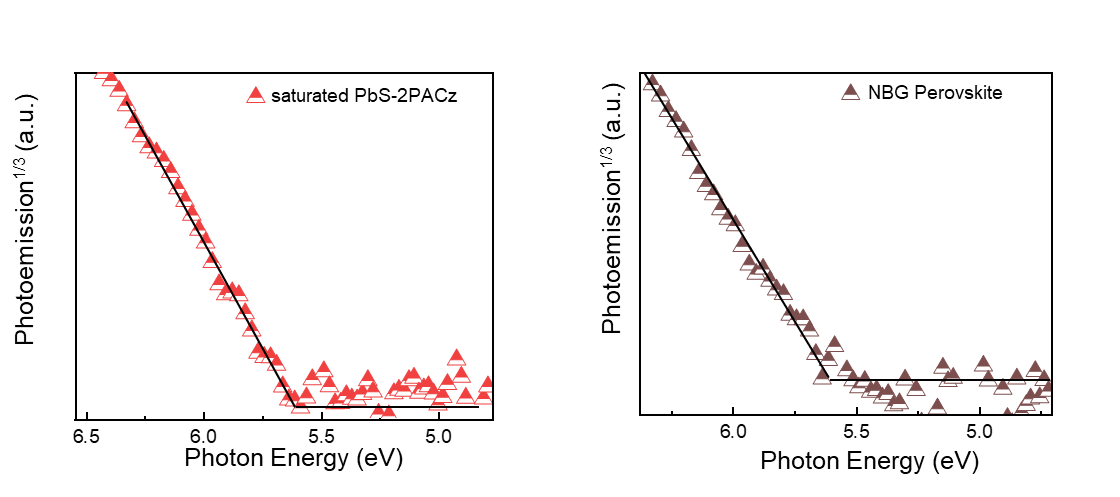


**Figure S13.** APS profiles for determining VBM of saturated PbS-2PACz and NBG perovskites.


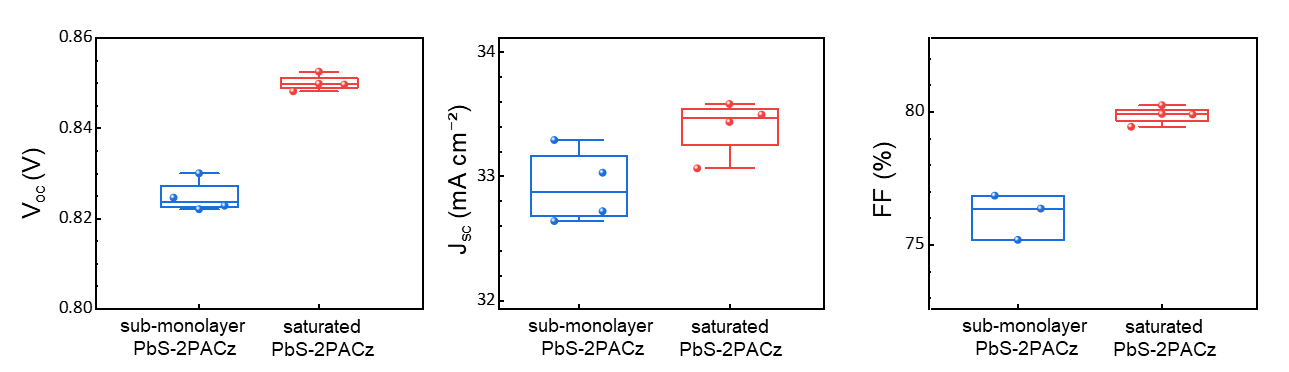


**Figure S14.** Box plots comparing key photovoltaic parameters for NBG PSCs with sub-monolayer PbS-2PACz and saturated PbS-2PACz.


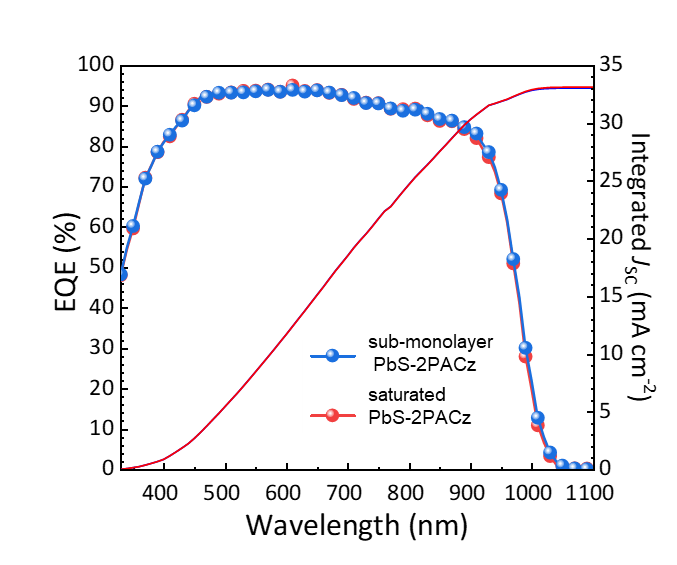


**Figure S15.** External quantum efficiency (EQE) spectra of NBG PSCs with sub-monolayer PbS-2PACz and saturated PbS-2PACz.


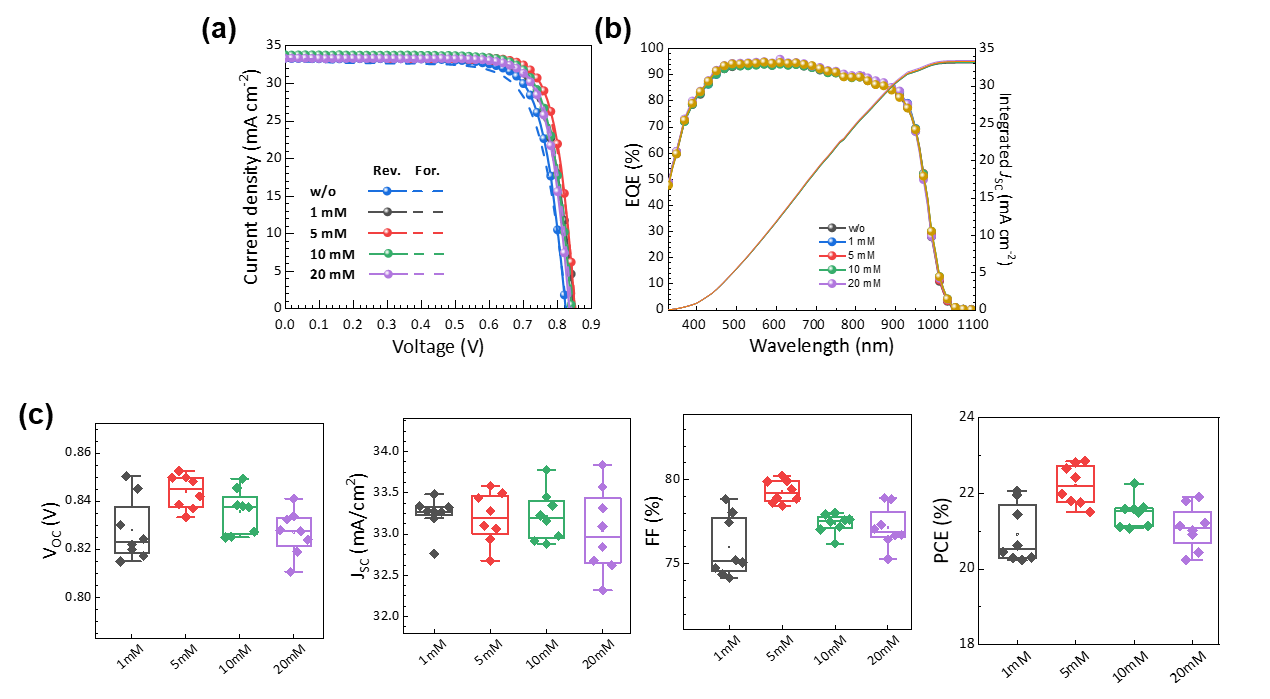


**Figure S16.** (a) *J‒V* curves and (b) EQE spectra of NBG PSCs with varying 2PACz solution concentrations (0–20 mM) on sub-monolayer PbS-2PACz film. (c) Box charts of photovoltaic parameters of NBG PSCs with varying 2PACz concentrations (0–20 mM) on PbS 2PACz film.


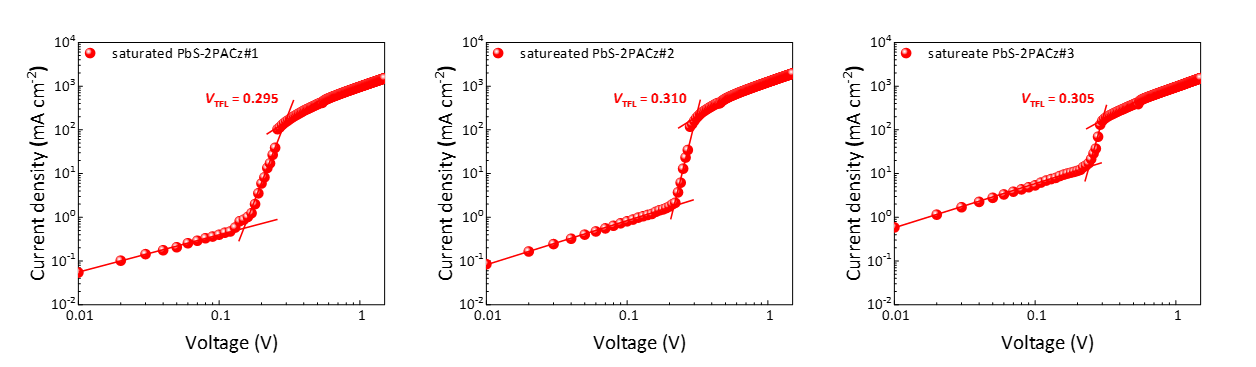


**Figure S17.** SCLC measurement for saturated PbS-2PACz hole-only devices, showing reduced *V*_TFL_ and trap density, indicative of improved interfacial passivation through the additional incorporation of 2PACz molecules on the PbS surface.


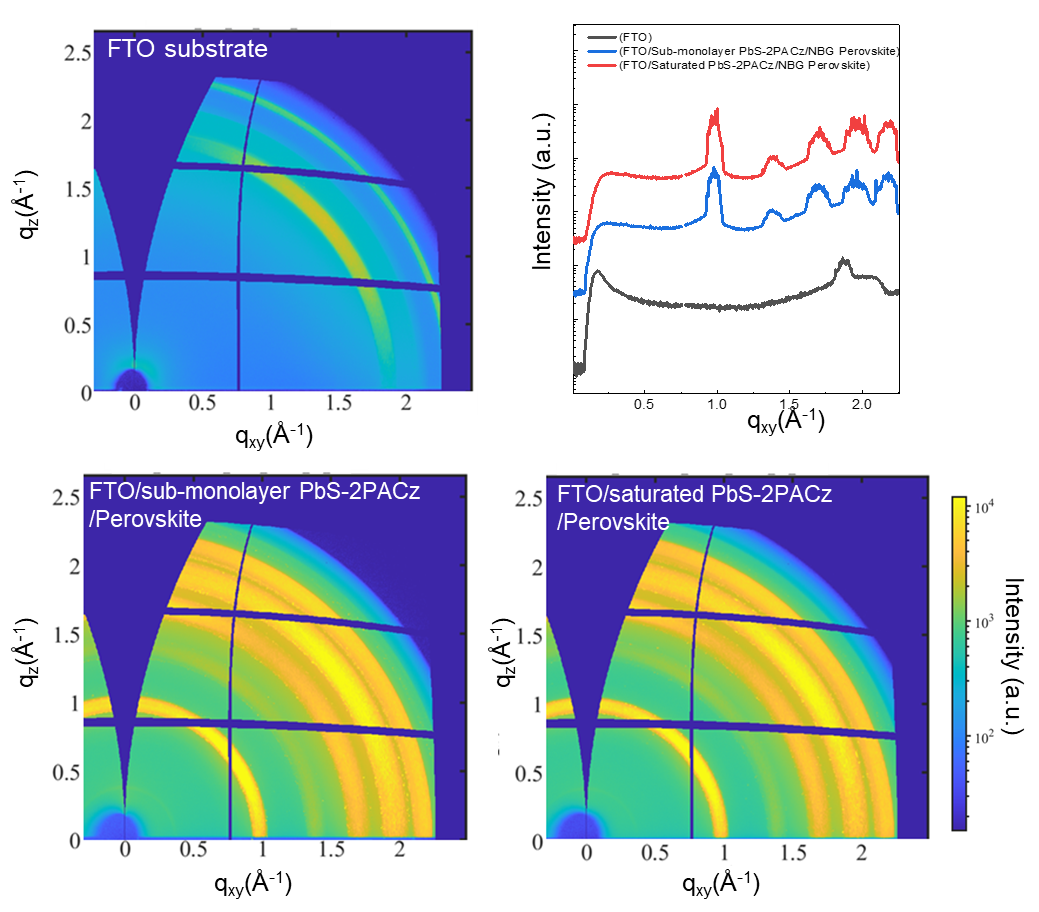


**Figure S18.** GIWAXS patterns of perovskite films deposited on sub-monolayer PbS-2PACz and saturated PbS-2PACz. These results indicate that the crystallinity of the perovskite remains unaffected by the presence of sub-monolayer PbS-2PACz or saturated PbS-2PACz, suggesting that modification did not disrupt the crystalline structure of the perovskite layer.


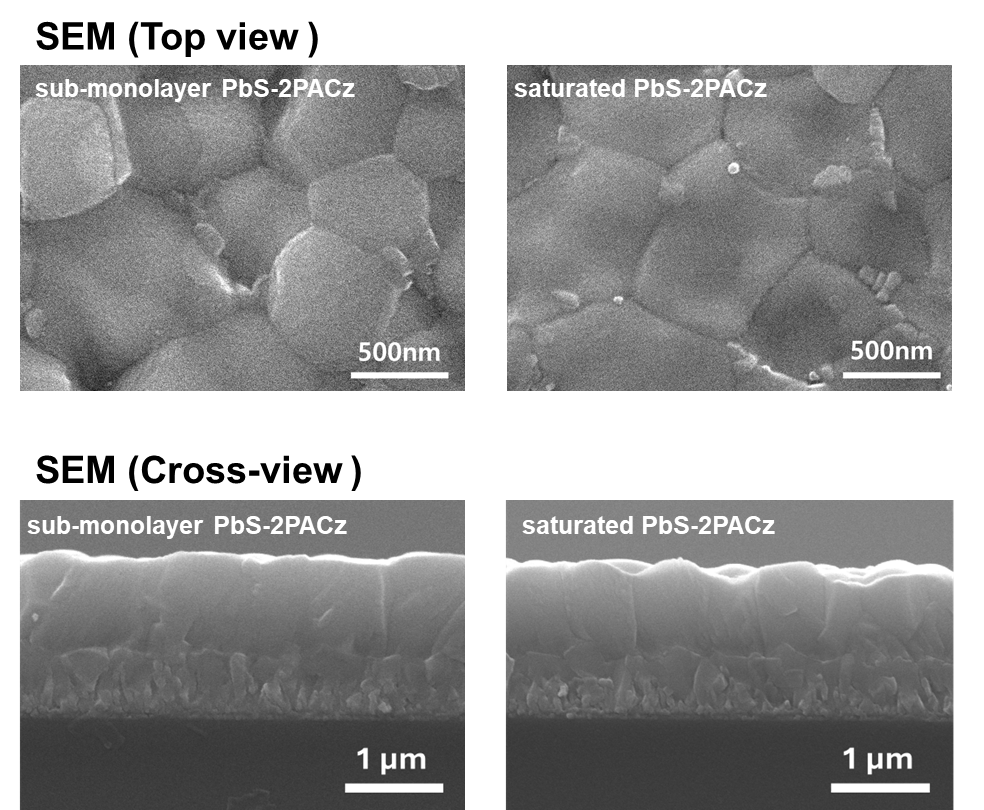


**Figure S19.** SEM images of perovskite films on sub-monolayer PbS-2PACz and saturated PbS-2PACz showing top and cross-sectional views. The results indicate negligible differences in the crystallinity and grain size of the perovskite under both conditions.


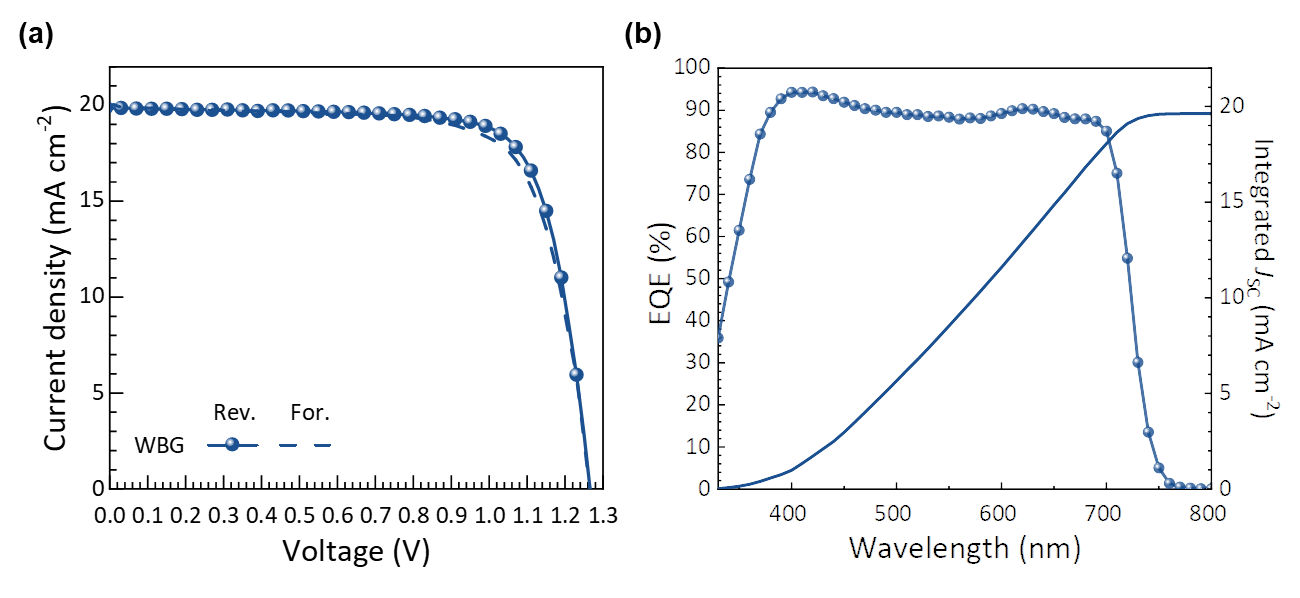


**Figure S20.** (a) J–V curves and (b) EQE spectra of wide-bandgap PSCs (Cs_0.2_FA_0.55_MA_0.25_Pb(I_0.85_Br_0.15_)_3_, bandgap: 1.71eV) with saturated PbS-2PACz as HTL


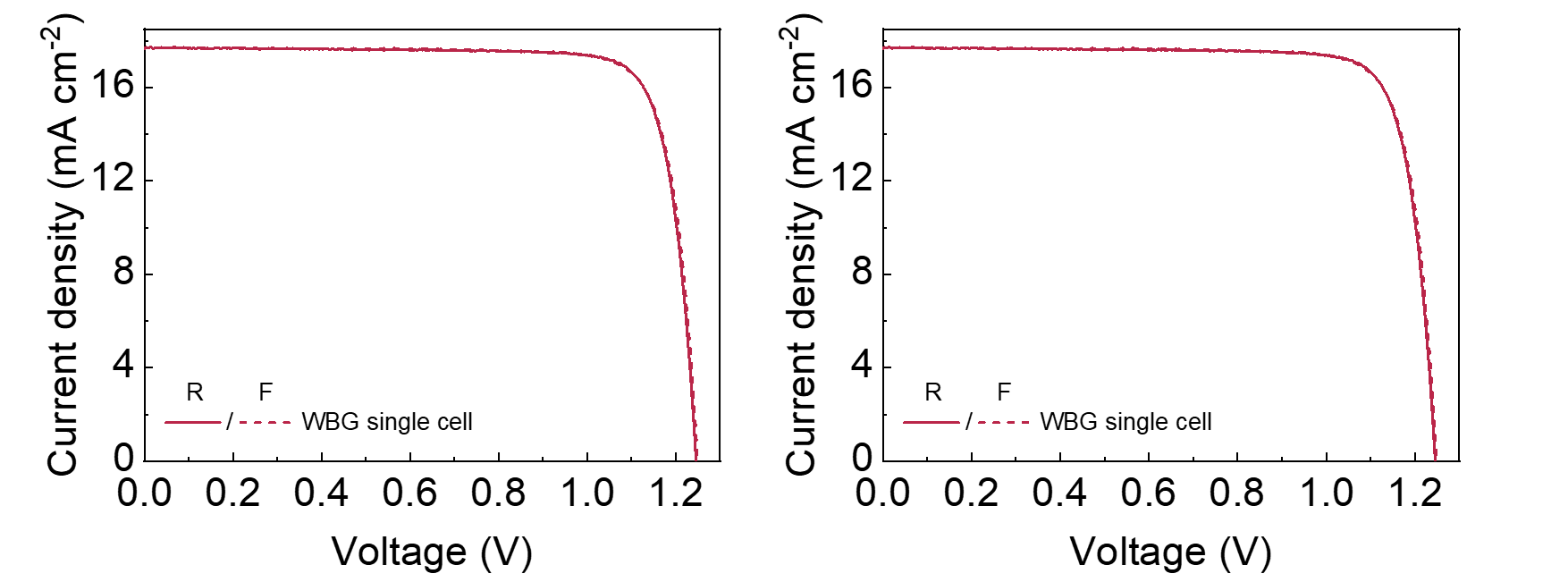


**Figure S21** J–V curves of wide-bandgap PSCs (FA₀.₆MA₀.₄Pb(I₀.₆Br₀.₄)₃-based front subcell).

| **HTL** | **τ_1_ (ns)** | **A1 (%)** | **τ_2_ (ns)** | **A2 (%)** | **τ_avg_ (ns)** |
| --- | --- | --- | --- | --- | --- |
| PEDOT:PSS | 135.48 ± 4.24 | 37.06 | 560.54 ± 7.62 | 62.94 | 403.01 |
| PbS-2PACz | 31.12 ± 0.50 | 48.05 | 114.24 ± 0.92 | 51.95 | 74.30 |

**Table S1.** TR-PL lifetimes of perovskite films on PEDOT:PSS and PbS-2PACz

| **HTL** | ***V_oc_***  **(V)** | **J_sc_**  **(mA/cm^2^)** | **FF**  **(%)** | **PCE(%)** |
| --- | --- | --- | --- | --- |
| PEDOT:PSS | 0.79 | 29.52 | 78.85 | 18.40 |
| 2PACz | 0.84 | 33.07 | 76.41 | 21.32 |
| PbS-2PACz | 0.82 | 33.29 | 76.85 | 21.05 |

**Table S2.** Photovoltaic performance metrics of NBG PSC devices with PEDOT:PSS, 2PACz, and PbS-2PACz

| **Components**  **of Pb 4f_7/2_** | **Binding energy (eV)** | **sub-monolayer**  **PbS-2PACz**  **(% Area)** | **saturated**  **PbS-2PACz**  **(% Area)** |
| --- | --- | --- | --- |
| Pb-S | 137.8 | 61 | 28 |
| Pb-O | 138.9 | 39 | 71 |

**Table S3.** XPS binding energies and relative peak areas (%) of Pb 4f7/2 components for sub-monolayer PbS-2PACz and saturated PbS-2PACz

| **HTL** | **τ_1_ (ns)** | **A1 (%)** | **τ_2_ (ns)** | **A2 (%)** | **τ_avg_ (ns)** |
| --- | --- | --- | --- | --- | --- |
| saturated  PbS-2PACz | 13.13 ± 0.13 | 67.83 | 51.60 ± 0.54 | 32.17 | 25.51 |

**Table S4.** TR-PL lifetimes of perovskite films on saturated PbS-2PACz

| **Variable** |  | ***V_oc_***  **(V)** | ***J_sc_***  **(mA/cm^2^)** | **FF** | **PCE**  **(%)** | **Integrated**  ***J*_SC_**  **(mA cm^-2^)** |
| --- | --- | --- | --- | --- | --- | --- |
| WBG | R | 1.265 | 19.84 | 76.21 | 19.13 | 19.62 |
|  | F | 1.265 | 19.92 | 73.40 | 18.49 |  |

**Table S5.** Photovoltaic performance metrics of wide-bandgap PSCs (Cs_0.2_FA_0.55_MA_0.25_Pb(I_0.85_Br_0.15_)_3_, bandgap: 1.71eV) with saturated PbS-2PACz as HTL

| **Variable** |  | ***V_oc_***  **(V)** | ***J_sc_***  **(mA/cm^2^)** | **FF** | **PCE**  **(%)** | **HF**  **(%)** |
| --- | --- | --- | --- | --- | --- | --- |
| WBG | R | 1.246 | 17.53 | 83.20 | 18.17 | 99.61 |
|  | F | 1.249 | 17.48 | 82.88 | 18.10 |  |

**Table S6.** Photovoltaic performance metrics of wide-bandgap PSCs (FA₀.₆MA₀.₄Pb(I₀.₆Br₀.₄)₃-based front subcell).

**Equation S1**

$J={9\varepsilon_{0}\varepsilon_{r}\mu V^{2}}/{8d^{3}}$

Here, *J* represents the current density, *V* is the applied voltage, *d* denotes the thickness of the quantum dot layer, *μ* is the charge carrier mobility, *ε_r_* is the relative dielectric constant of the transport medium, and *ε_0_*​ is the permittivity of free space.

**Equation S2**

$$V_{TFL}=(2\varepsilon\varepsilon_{r} N_{trap})/(qL^{2} )$$

Here, *ε_r_* is the relative dielectric constant of the NBG perovskite (*ε_r_* = 28.8), *ε* is the vacuum permittivity (8.854 × 10^-12^ F/m), *V_TFL_* is the onset voltage of the trap-filled limit region, *q* is the elemental charge, and *L* is the thickness of the perovskite film. *N_trap_* is the trap density (sub-monolayer PbS-2PACz: 0.94 × 10^15^ cm^-3^, and saturated PbS-2PACz: 0.87 × 10^15^ cm^-3^).
